# Serum Metabolome and Lipidome Changes in Adult Patients with Primary Dengue Infection

**DOI:** 10.1371/journal.pntd.0002373

**Published:** 2013-08-15

**Authors:** Liang Cui, Yie Hou Lee, Yadunanda Kumar, Fengguo Xu, Kun Lu, Eng Eong Ooi, Steven R. Tannenbaum, Choon Nam Ong

**Affiliations:** 1 Interdisciplinary Research Group in Infectious Diseases, Singapore-MIT Alliance for Research & Technology (SMART), Singapore; 2 Saw Swee Hock School of Public Health, National University of Singapore, Singapore; 3 Departments of Biological Engineering and Chemistry, Massachusetts Institute of Technology, Cambridge, Massachusetts, United States of America; 4 DUKE-NUS Graduate Medical School, Singapore; 5 NUS Environment Research Institute, Singapore; Florida Gulf Coast University, United States of America

## Abstract

**Background:**

Dengue virus (DENV) is the most widespread arbovirus with an estimated 100 million infections occurring every year. Endemic in the tropical and subtropical areas of the world, dengue fever/dengue hemorrhagic fever (DF/DHF) is emerging as a major public health concern. The complex array of concurrent host physiologic changes has hampered a complete understanding of underlying molecular mechanisms of dengue pathogenesis.

**Methodology/Principle Findings:**

Systems level characterization of serum metabolome and lipidome of adult DF patients at early febrile, defervescence, and convalescent stages of DENV infection was performed using liquid chromatography- and gas chromatography-mass spectrometry. The tractability of following metabolite and lipid changes in a relatively large sample size (*n* = 44) across three prominent infection stages allowed the identification of critical physiologic changes that coincided with the different stages. Sixty differential metabolites were identified in our metabolomics analysis and the main metabolite classes were free fatty acids, acylcarnitines, phospholipids, and amino acids. Major perturbed metabolic pathways included fatty acid biosynthesis and *β*-oxidation, phospholipid catabolism, steroid hormone pathway, etc., suggesting the multifactorial nature of human host responses. Analysis of phospholipids and sphingolipids verified the temporal trends and revealed association with lymphocytes and platelets numbers. These metabolites were significantly perturbed during the early stages, and normalized to control levels at convalescent stage, suggesting their potential utility as prognostic markers.

**Conclusions/Significance:**

DENV infection causes temporally distinct serum metabolome and lipidome changes, and many of the differential metabolites are involved in acute inflammatory responses. Our global analyses revealed early anti-inflammatory responses working in concert to modulate early pro-inflammatory processes, thus preventing the host from development of pathologies by excessive or prolonged inflammation. This study is the first example of how an omic- approach can divulge the extensive, concurrent, and dynamic host responses elicited by DENV and offers plausible physiological insights to why DF is self limiting.

## Author Summary

Dengue virus is the most widespread arbovirus and a major public health threat in the tropical and subtropical areas of the world. As yet, little is known about the molecular mechanisms underlying infection, and there is no specific treatment or vaccine that is currently effective against the disease. Metabolomics and lipidomics provide global views of metabolome and lipidome landscapes and implicate metabolic to disease phenotype. We performed serum metabolic and lipidomic profiling on a cohort of dengue patients with three sampling time points at early febrile, defervescence, and convalescent stages via mass spectrometry-based analytical platforms. Compared with healthy subjects, approximately two hundred metabolites showed significant difference in dengue patients, and 60 were identified. This study revealed that in primary dengue infection, the host metabolome is tightly regulated, with active, early anti-inflammatory processes modulating the pro-inflammatory processes, suggesting the self-limiting phenotype of dengue fever. Major perturbed metabolic pathways included fatty acid biosynthesis, fatty acid *β*-oxidation, phospholipid catabolism, steroid hormone pathway, etc. This represents a first report on the characterization of the serum metabolome and significantly advances our understanding on host and dengue virus interactions. These differential metabolites have the potential as biomarkers for disease monitoring and evaluation of therapeutic interventions.

## Introduction

Dengue virus (DENV) is a member of the *Flavivirus* genus of the *Flaviviridae* family and is classified into four distinct serotypes. It is estimated that 100 million dengue infections occur worldwide every year, ranging from acute dengue fever (DF) to life-threatening dengue haemorrhagic fever/dengue shock syndrome (DHF/DSS) [1], [2]. Dengue is emerging as a global health concern with the annual average number of DF/DHF cases reported to World Health Organization increasing dramatically in recent years. Furthermore, DENV has spread widely, and all four serotypes are now circulating in Asia, Africa and the Americas [3].

DF is generally self-limiting, but its symptoms can be debilitating and cause considerable incapacitating morbidity, which have a significant health and economic toll in the society. In a small percentage of patients, DF evolves to the more severe forms of DHF and DSS, which are characterized by abnormal hemostasis, vascular leakage and liver damage. There is currently no specific antiviral therapy or vaccine available for DF or DHF/DSS. Furthermore, the underlying molecular mechanisms of DENV pathogenesis are still unclear and why DF resolves in time is largely neglected. Therefore, the pathological differences between the severe DHF/DSS and the mild, self-limiting, DF suggest differential virus-host interactions in the susceptibility to the disease. Both viral and host immune factors seem to be involved, but the role of each is not fully understood [4]. Meanwhile, the lack of an appropriate animal model significantly increases the challenges in the study of DENV pathogenesis.

Metabolomics and lipidomics are rapidly emerging fields of ‘omics’ research that aim to study the global changes of small molecule metabolites and lipids in biological systems in response to biological stimuli or perturbations [5]. Metabolites are the end products of cellular regulatory processes, forming a link between molecular changes and phenotype, and therefore reflect the physiological state of a cell, tissue or organism at a point in time. Lipidomics covers the subset of lipid constituents, which are known to be involved in signalling and infection biology. Cellular homeostasis is interrupted under disease conditions, and the human body would attempt to maintain a basal, internal environment by increasing or decreasing levels of certain endogenous metabolites. Thus, by providing a snapshot of the metabolic status of an organism, metabolomics holds the promise of finding metabolic pathways related to disease processes [6], [7]. Metabolomics has been applied to infectious diseases to study host-pathogen interactions [8], [9], [10]. Similarly, there has been enormous interest in how lipids and their metabolism influence *Flaviviridae* viral infection, especially from the perspective of microbe lipid usage for virus entry, replication and release [11], [12], [13]. As we redefine the manner in which we understand the complex and dynamic virus-host interactions of dengue infection *in vivo*, it necessitates capturing the global changes rather than isolated ones. However, few omics studies exist that describe how the human host response biochemically and physiologically during DENV and other flavivirus infection and how it resolves without intervention [14], [15]. Therefore we turned our attention to examine serum metabolites and lipids using a comprehensive approach to globally capture the human responses to DF.

In our previous study, a systematic characterization of serum cytokines, proteome, and markers of macrophage and neutrophil activity was reported from a subset of longitudinally enrolled adult patients with primary dengue infections [16]. In the present study, both untargeted metabolomics and targeted lipidomics on the same serum sample set were conducted with the aim to identify metabolic pathways linked to disease progression and understand the mechanisms of DENV infection. Our results showed that DENV infection caused significant serum metabolome and lipidome-wide changes in DF patients. Sixty differential metabolites were identified in metabolomics analysis and detailed examination of metabolic pathways changes revealed that in primary DENV infections, acute inflammatory responses were quickly met with active pro-resolution counterbalances to restore homeostasis. Our studies also implicated the plausible association of sphingomyelins with CD8+ lymphocyte activation during the early febrile period, and plasmalogen phosphatidylethanolamines and lysophosphatidylethanolamines with platelet numbers during the later phases of defervescence and convalescence. We propose that early widespread host changes in the metabolome and lipidome play an important physiological component in defining the self-limiting outcome of DF.

## Materials and Methods

### Ethics statement

The early dengue infection and outcome (EDEN) study is a multi-center longitudinal study, which prospectively recruits and follows-up adult dengue patients in Singapore, through early febrile, defervescence as well as convalescence stages of the disease [17]. Enrolment of all eligible individuals was based on written informed consent and the protocols were approved by the National Healthcare group (DSRB B/05/013). All samples were anonymized.

### Clinical samples

The details of patient recruitment, sample collection and the study protocols of EDED study have been described earlier [18]. In brief, adult patients (>21 years) presenting with acute onset fever (≥38.0°C for less than 72 hours) without rhinitis or other clinical alternatives were included in the study (Visit 1; febrile). Initial dengue diagnosis was made by real time RT-PCR and followed by serology and subsequent serotyping by virus isolation and immunofluorescence using serotype specific monoclonal antibodies (ATCC: HB46-49). Venous blood samples were also collected at fever day 4 to 7 (Visit 2; defervescence) and weeks 3 to 4 (Visit 3; convalescence), aliquoted and frozen at −80°C. ‘Fever day’ here refers to number of days post onset of fever. Classification of DF was made based on the guidelines provided by the World Health Organisation [19]. In brief, acute febrile patients positive for dengue with one or more of the following symptoms: headache, retro orbital pain, myalgia, rash, leucopenia, hemorrhage were classified as DF. Of the 133 dengue patients that were finally enrolled in this study (September 2005–October 2006), 44 DF patients tested negative for dengue IgG antibodies in the acute sera, using a commercially obtained ELISA (PanBio, Brisbane, Australia). These patients were deemed to have primary DENV infection, all of which were included in this study. A detailed hematological and virological analysis was also performed. Additionally we used serum samples from 50 asymptomatic age-matched healthy subjects participated during a hospital staff annual examination as controls (**Table 1**). This study was approved by the National University of Singapore Institutional Review Board and samples were collected with individual informed written consents.

**Table 1:** Demographics of healthy subjects and dengue patients enrolled in this study.

Patient Groups	Serotype	Age	Gender	Blood sampling time*
DF (*n* = 44)	D1 (54.5%)	39.0±13.0	27 (male)	46±20 hours (Visit 1)
	D2 (2.3%)		17 (female)	73±31 hours (Visit 2)
	D3 (43.2%)			16±5 days (Visit 3)
	D4 (0)			
Controls (*n* = 50)	NA	40.0±18.0	14 (male)	NA
			36 (female)	

* Average time from fever to phlebotomy (Visit 1), between Visits 1 & 2 (Visit 2) and between Visits 2 & 3 (Visit-3).

### Serum sample preparation

For metabolomics analysis, a volume of 50 µL from each serum sample was thawed at 4°C. Serum proteins were precipitated with 200 µL ice-cold methanol, which contained 10 µg/mL 9-fluorenylmethoxycarbonyl-glycine as an internal standard. After vortexing, the mixture was centrifuged at 16,000 rpm for 10 minutes at 4°C and the supernatant was divided into two parts, one for liquid chromatography/mass spectrometry (LC-MS) analysis and the other for gas chromatography/mass spectrometry (GC-MS) analysis. All samples were kept at 4°C and analyzed within 48 h. In order to prevent batch effect, the assays were conducted in random manner. Quality control (QC) samples were prepared by mixing equal amounts of serum samples from 10 healthy subjects and 10 DF patients at all the three time points.

For lipidomics analysis, due to limited sample availability, sera from three patients at each visit were randomly pooled (14 pools total). Lipids were extracted from serum using a modified Bligh and Dyer method [20]. Briefly, 900 µL of chloroform-methanol, 1∶2 (v/v) was added to 100 µL serum. After 20 min vortexing and incubation at 4°C, 300 µL of chloroform and 300 µL of ddH_2_O were added to the mixture and centrifuged at 9000 rpm, 4°C for 2 min. Lipids were then recovered from the lower organic phase after centrifugation. Subsequently, 500 µL of chloroform was added, vortexed at 4°C for 20 min. After centrifugation, lipids were recovered from the organic phase and combined with the previous fraction. The lipid extracts were vacuum-dried, stored at −80°C and analyzed within a week.

### Metabolomics analysis by LC-MS

The supernatant fraction from sample preparation step was analyzed using Agilent 1290 ultrahigh pressure liquid chromatography system (Waldbronn, Germany) equipped with a 6520 Q-TOF mass detector managed by a MassHunter workstation. The column used for the separation was an Agilent rapid resolution HT Zorbax SB-C18 (2.1×50 mm, 1.8 µm; Agilent Technologies, Santa Clara, CA, USA). The oven temperature was set at 50°C. The gradient elution involved a mobile phase consisting of (A) 0.1% formic acid in water and (B) 0.1% formic acid in methanol. The initial condition was set at 5% B. A 7 min linear gradient to 70% B was applied, followed by a 12 min gradient to 100% B which was held for 3 min, then returned to starting conditions over 0.1 min. Flow rate was set at 0.4 ml/min, and 5 µL of samples was injected. The electrospray ionization mass spectra were acquired in both positive and negative ion mode. Mass data were collected between m/z 100 and 1000 at a rate of two scans per second. The ion spray voltage was set at 4,000 V, and the heated capillary temperature was maintained at 350°C. The drying gas and nebulizer nitrogen gas flow rates were 12.0 L/min and 50 psi, respectively. Two reference masses were continuously infused to the system to allow constant mass correction during the run: m/z 121.0509 (C_5_H_4_N_4_) and m/z 922.0098 (C_18_H_18_O_6_N_3_P_3_F_24_). The stability of the LC-MS method was examined and evaluated by a subset of peaks covering a range of masses, intensities, and retention times across the QC samples (**Table S1**). Variations of retention time and m/z values of the peaks were less than 0.2 min and 10 mDa, respectively, and the relative standard deviations of peak areas were below 20%, indicating good reproducibility and stability of the chromatographic separation and mass accuracy of mass measurement during the whole sequence.

### Metabolomics analysis by GC-MS

100 µL supernatant fraction from sample preparation step was dried under nitrogen and derivatised with 150 µL methoxamine (50 µg/mL in pyridine, 37°C×2 h) followed by 150 µL *N*-methyl-trimethylsilyl-trifluoroacetamide (37°C×16 h). After centrifugation (4°C, 6000 rpm×1 min), the supernatant fraction was injected into GC-MS. The derivatised sample (1.0 µL) was introduced by splitless injection with an Agilent 7683 Series autosampler into an Agilent 6890 GC system equipped with a fused-silica capillary column HP-5MSI (30 m×0.25 mm i.d., 0.25 µm film thickness) as reported previously [21]. The inlet temperature was set at 250°C. Helium was used as the carrier gas at a constant flow rate of 1.0 ml/min. The column effluent was introduced into the ion source of an Agilent Mass selective detector. The transfer line temperature was set at 280°C and the ion source temperature at 230°C. The mass spectrometer was operated in electron impact mode (70 eV). Data acquisition was performed in full scan mode from m/z 50 to 550 with a scan time of 0.5 sec. The compounds were identified by comparison of mass spectra and retention time with those of reference standards, and those available in libraries (NIST 0.5). A summary of the workflow utilized in metabolomics study is shown in **Figure S1**.

### Lipidomics analysis by LC-MS/MS

To facilitate interrogation of the serum lipidome in a high-throughput, targeted and accurate fashion, we employed LC-MS/MS in Multiple Reaction Monitoring (MRM) mode to survey more than 200 individual glycerophospholipids and sphingolipids simultaneously at linear dynamic ranges of 0.1 to 100 ng/mL. Prior to ESI-LC-MS/MS, each lipid extract was reconstituted in 400 µL of chloroform-methanol (1∶1 v/v). LC-MS/MS via Triple Quadrupole 6460 with Jet Stream™ (Agilent Technologies) was used for the quantification of glycerophospholipids and sphingolipids (total >200 molecular species). C-18 reversed phase LC (Zorbax Eclipse 2.1×50 mm I.D., 1.8 µm; Agilent Technologies) was used to separate 5 µL reconstituted lipids at 400 µL/min before entering the mass spectrometer. The column thermostat and autosampler temperatures were maintained at 40°C and 6°C, respectively. The optimized mobile phase consisted of 5 mM ammonium acetate in water (solvent A) and 5 mM ammonium acetate in methanol (solvent B). The gradient of the samples was: 0–2 min, 60–100% B; 2–7 min, maintained at 100% B; 7–9 min, 100–60% B and 9–11 min, 60% for column re-equilibration before the next injection. Positive ionization mode [M+H]^+^ MRM was based on product ion *m/z* 264.4 [sphingosine-H_2_O]^+^ and 266.4 [sphinganine-H_2_O]^+^ for ceramide (Cer), monohexosylceramide (MHexCer), lactosylceramide (LacCer) and ceramide-1-phosphate (C1P), and *m/z* 184.1 [phosphocholine]^+^ for sphingomyelin (SM). Negative ionization [M-H]^−^ MRM mode based on specific parent and product ion pairs was employed for phosphatidic acid (PA), phosphatidylinositol (PI), lysophosphatidylinositol, phosphatidylethanolamine (PE), lysophosphatidylethanolamine (lysoPE), plasmalogen phosphatidylethanolamine (pPE), phosphatidylglycerol (PG), phosphatidylserine (PS) and lysophosphatidylserine (lysoPS) for identification and quantification of the above glycerophospholipids [22]. The optimized lipid-class dependent mass spectrometry parameters are shown in ****. Known concentrations (20–100 ng/mL) of non-naturally occurring internal standards, namely Cer d18:1/17:0, GlcCer d18:1/8:0, LacCer d18:1/8:0, SM d18:1/12:0, C1P d18:1/17:0, PA 14:0, PC 14:0, PE 14:0, PG 14:0, PS 14:0 and PI 14:0; Avanti Polar Lipids, Alabaster, Al) were verified of their low abundance in sera, spiked into extracted samples and used in absolute quantification. The linear dynamic ranges, limits of detection, limits of quantification and relative standard deviation of the various lipids are shown in **Table S3**. The effect of acyl length on signal intensity [23], [24] was compensated by adjusting the collision energy and fragmentor voltage (**Table S2**). A summary of the workflow utilized in the lipidomics study is shown in **Figure S2**.

### Data analysis

For metabolomics analysis, raw spectrometric data were converted to mzData (LC-MS/MS) and NetCDF (GC-MS) formats via Masshunter (Agilent, US) and input to open-source software MZmine 2.0 for peak finding, peak alignment and peak normalization across all samples [25]. The MZmine 2.0 report table was exported into SIMCA-P software (version 11.0; Umetrics AB, Umea, Sweden) for multivariate data analysis. Data sets were mean-centered and pareto-scaled to adjust the importance of high and low abundance metabolites to an equal level before carrying out statistical analyses. Unsupervised multivariate analysis principle component analysis (PCA) was first performed on LC-MS data as an unbiased statistical method to observe intrinsic trends within healthy controls and DF patients at different visits. To achieve the maximum separation among the groups, supervised multivariate analysis orthogonal partial least-squares discriminant analysis (OPLS-DA) was sequentially applied. Potential differential metabolites were selected according to the Variable Importance in the Projection (VIP) values and the variables with VIP>1 were considered to be influential for the separation of samples in OPLS-DA analysis. In addition, the Kruskal-Wallis test was performed to determine if the differential metabolites obtained from OPLS-DA modelling were statistically significant (*p*<0.05) among groups at the univariate analysis level.

For lipidomics analysis, undetectable lipids were replaced with 1/10 of the lowest non-zero value measured. In cases where 70% of data are missing from patients, lipids were excluded. Missing data represents 1.8% of the data. In total 207 lipids remained for further analysis. Comparisons between groups were achieved using non-parametric analysis namely two-tailed Mann-Whitney tests and Kruskal-Wallis tests with Dunn's *post hoc* analysis. Repeated one-way ANOVA was used for paired analysis with the Bonferroni Multiple Comparison *post hoc* Test.

### Compound identification and pathways analysis

The structure identification of the differential metabolites was based on a previously described strategy [26]. The identification process is illustrated here with hexanoylcarnitine, one of the ten differential acylcarnitines. First, the element composition C_13_H_25_NO_4_ of the *m/z* 260.18 ion was calculated based on the exact mass, the nitrogen rule and the isotope pattern by Masshunter software from Agilent (**Figure S3A**). Then, the elemental composition and exact mass were used for open source database searching, including LIPIDMAPS (http://www.lipidmaps.org/), HMDB (http://www.hmdb.ca/), METLIN (http://metlin.scripps.edu/) and MassBank (http://www.massbank.jp/). Next, MS/MS experiments were performed to obtain structural information via the interpretation of the fragmentation pattern of the metabolite (**Figure S3B**). In the case of acylcarnitines, *m/z* 85.0 is the characteristic fragment ion in the MS/MS spectrum [27]. The MS/MS spectra of possible metabolite candidates in the databases were also searched and compared. As a result, the metabolite was identified as hexanoylcarnitine, which was finally confirmed by comparison with the standard (**Figure S3C**). Both MetaboAnalyst [28] and Ingenuity Pathway Analysis software (IPA, www.ingenuity.com) were used to identify relevant pathways.

## Results

### Global metabolome changes with dengue fever

**Figure 1:**
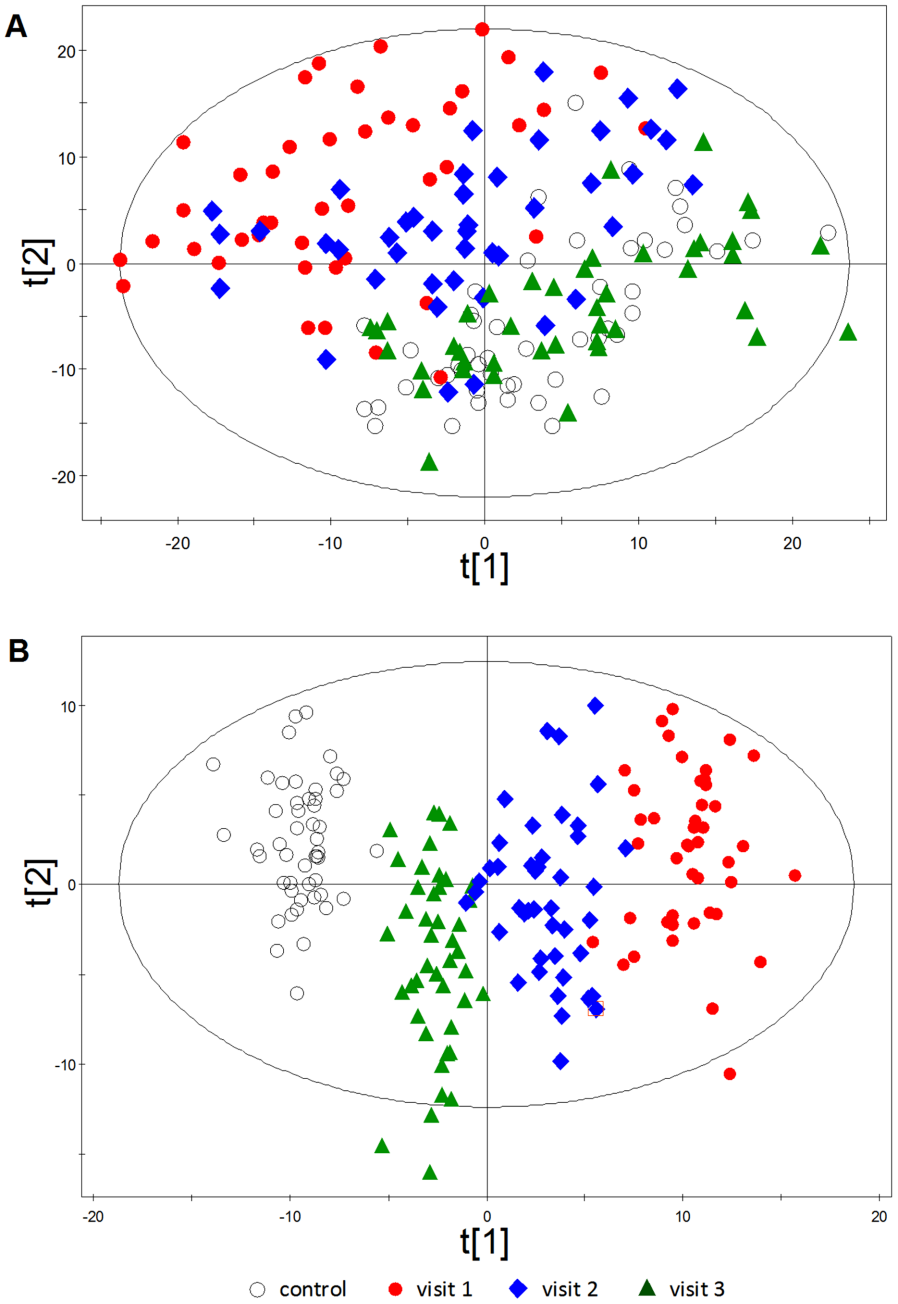
PCA (A) and OPLS-DA (B) score plots of dengue fever based on metabolomics data. The principle component analysis (PCA) and orthogonal partial least-squares discriminant analysis (OPLS-DA) models were constructed using LC-MS/MS metabolomics data from healthy subjects and dengue patients at three Visits.

In order to obtain a high level snapshot of metabolite flux at the different stages of dengue infection represented in our clinical samples, we examined variances in our data both by principal component analysis (PCA) as well as OPLS-DA. The PCA score plot (**Figure 1A**) indicates that the metabolome changes in DF patients are reversible. The most predominant metabolome changes in DF patients, as compared with healthy controls, happened at early febrile stage (Visit 1), followed by the defervescence stage (Visit 2) and finally returned to the control levels at the convalescence stage (Visit 3). This indicates that the most intense responses of the DF patients to DENV infection happen within the first 72 hours upon infection, and homeostatic recovery appears to begin around the fourth day from the onset of fever. This, more or less, coincides with the course of viraemia, which has been observed to peak during early febrile phase and are generally undetectable during the defervescence stage. The duration of DF illness in the EDEN study is reported to be 7–14 days [18], and a majority of patients recover within three to four weeks as seen at Visit 3, which could explain the metabolome similarities between patients at Visit 3 and healthy controls in the PCA score plot. In addition, PCA did not reveal distinct metabolome profiles between DENV serotype 1 and DENV serotype 3. This suggests converging host responses between the two serotypes which resulted in DF. While host molecular changes may be present between infections with different DENV serotypes, a larger patient set will be required to tease out the significant subtle differences.

OPLS-DA, which is a more rigorous classification tool, revealed clearer segregation of the healthy controls and DF patients during the three visits (**Figure 1B**). The quality of the OPLS-DA model was evaluated by *R^2^* (Y) and *Q^2^* parameters, which indicated the fitness and prediction ability, respectively. By using four principle components, the OPLS-DA model showed a *R^2^* value of 0.95 and *Q^2^* value of 0.91, indicative of optimal performance of the model. The OPLS-DA was also applied to GC-MS data, which showed a *R^2^* value of 0.65 and *Q^2^* value of 0.60.

### Identification of significantly altered metabolites and pathways

**Figure 2:**
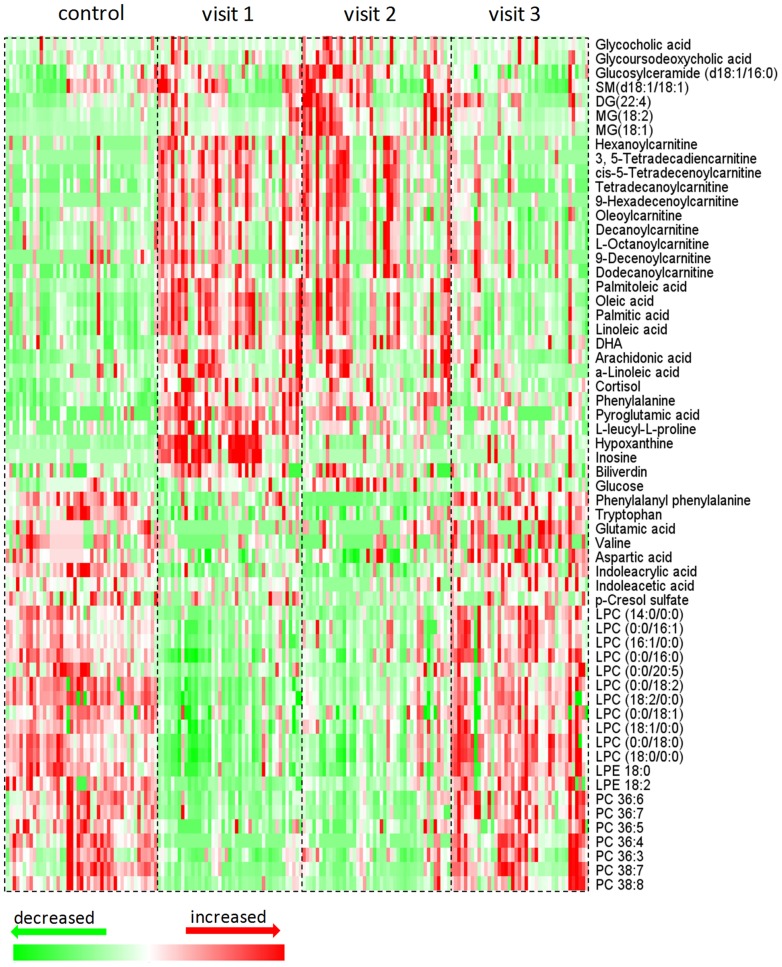
Heat map of identified differential metabolites from both LC-MS/MS and GC-MS analyses. Each row shows ion intensity for a specific metabolite after mean centering and unit variance scaling of the data. Each column shows the serum metabolic profiles of healthy subjects and dengue patients at three visits. * differential metabolites from GC-MS analysis.

Approximately 200 differential metabolites identified via both LC-MS/MS and GC-MS analyses were selected based on the criteria- VIP>1 in OPLS-DA analysis and *p*<0.05 in Kruskal-Wallis test (**Table S4, S5**). Sixty of these metabolites were structurally identified, with 56 through LC-MS/MS analysis, and 8 by GC-MS analysis, 4 of which were common to both these methods. These metabolites belonged to classes such as free fatty acid, acylcarnitine, phosphatidylcholine (PC), lysophosphatidylcholine (LysoPC), LysoPE, amino acid and derivative, sphingolipid, monoacylglyceride (MG), diacylglyceride (DG), purine, bile acid, bile pigment, steroid hormone, glucose and phenol sulfate (**Table S4**). Consistent with the PCA score plot (**Figure 1A**), the quantitative heatmap (**Figure 2**) of these differential metabolites revealed that the metabolome changes were reversible in DF patients. They were significantly perturbed during the early stages, and normalized to control levels at convalescent stage.

These differential metabolites show two contrasting time-course changes, an elevated and a decreased trend, at Visit 1 and Visit 2. Most of the metabolites have either the highest or the lowest levels at Visit 1, which is the acute stage of DENV infection, and gradually return to the control levels at Visit 3, indicating that they might serve as prognostic markers of the disease.

**Figure 3:**
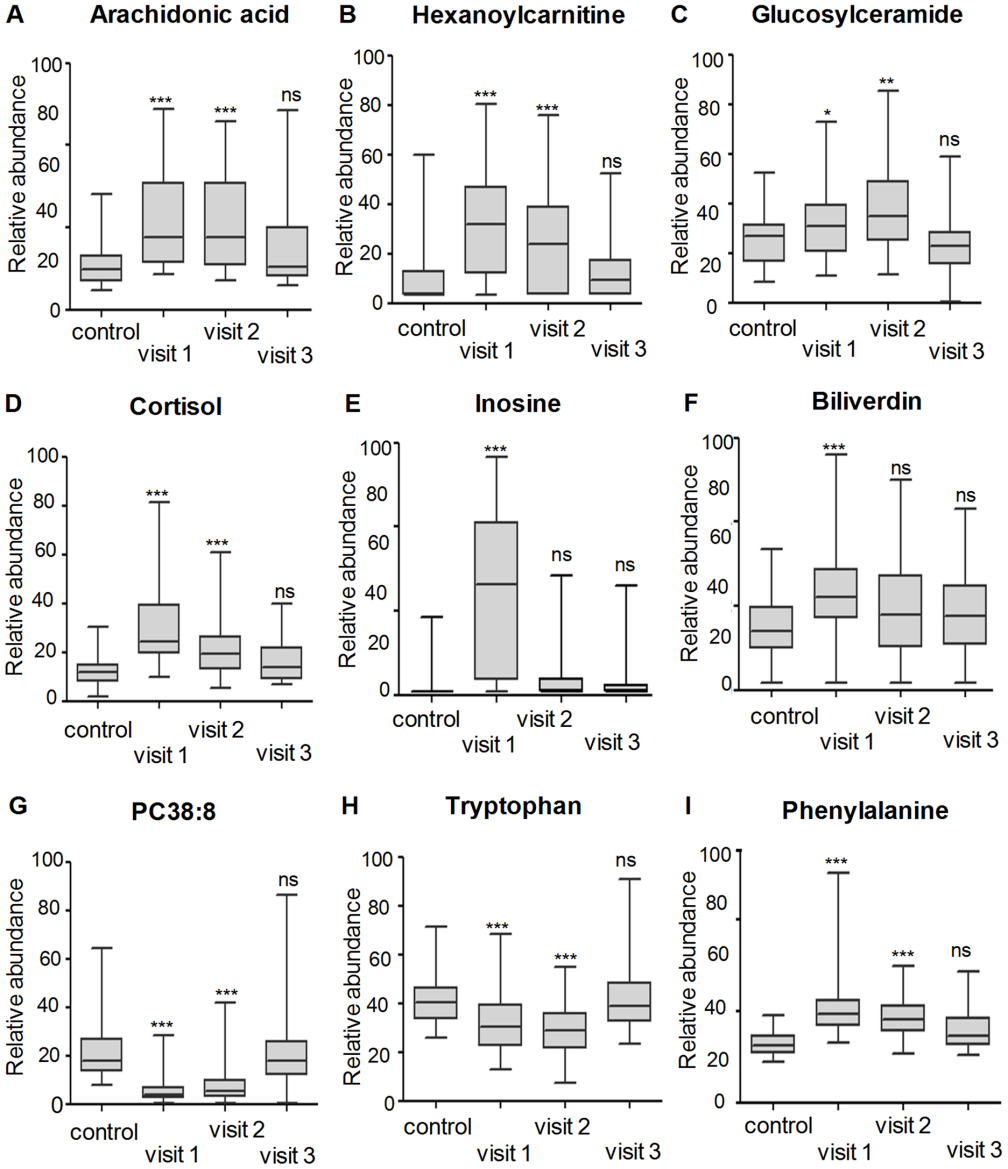
Typical change trends based on differential metabolite classes. **A.** elevated change trend of arachidonic acid (free fatty acid). **B.** elevated change trend of hexanoylcarnitine (acylcarnitine). **C.** elevated change trend of glucosylceramide (sphingolipid). **D.** elevated change trend of cortisol (steroid hormone). **E.** elevated change trend of inosine (purine). **F.** elevated change trend of biliverdine (bile pigment). **G.** decreased change trend of PC 38∶8 (phospholipid). **H.** decreased change trend of tryptophan (amino acid). **I.** elevated change trend of phenylalanine. Median-horizontal line in the box, The bottom and the top of the box are the 25^th^ and the 75^th^ percentiles, and the black band near the middle of the box is the median peak area of the metabolite. * *p*<0.05, ** *p*<0.01, *** *p*<0.001, ns, non-significant by test Kruskal-Wallis test. The statistical comparison was with control levels.

It is interesting that the metabolites within each metabolite class generally showed a similar change trend. Specifically, seven FFAs, including four polyunsaturated fatty acids (PUFAs): arachidonic acid (AA; 20:4*n*-6; **Figure 3A**), linoleic acid (18:2*n*-6), docosahexaenoic acid (DHA; 22:6*n*-3) and α-linoleic acid (18:3*n*-3), showed an elevated trend at Visit 1 and Visit 2. The PUFAs are released from cell-membrane phospholipids by various phospholipase enzymes, predominantly phospholipase A2, which is elevated in dengue patients [29]. Similarly, ten acylcarnitines, two sphingolipids (glucosylceramide and SM), two MG, one DG, one steroid hormone (cortisol), two purine nucleosides (hypoxanthine and inosine), one bile pigment (biliverdin), two conjugated bile acids (glycoursodeoxycholic acid and glycocholic acid), and glucose showed an increasing trend at Visit 1 and Visit 2 (**Figure 3B–3F**). Conversely, a total of twenty PCs, LPCs and lysoPEs showed a decreased trend (**Figure 3G**). Ten amino acids and derivatives, including tryptophan (**Figure 3H**), glutamic acid etc, also displayed a downward trend, with the exception of phenylalanine (**Figure 3I**), pyroglutamic acid, L-leucyl-L-proline, which showed an upward trend. The changes of amino acids observed are consistent with a previous report that described an overall decrease in plasma amino acid levels in dengue patients, with the exception of increased phenylalanine in the acute phase of DF patients [30].

**Figure 4:**
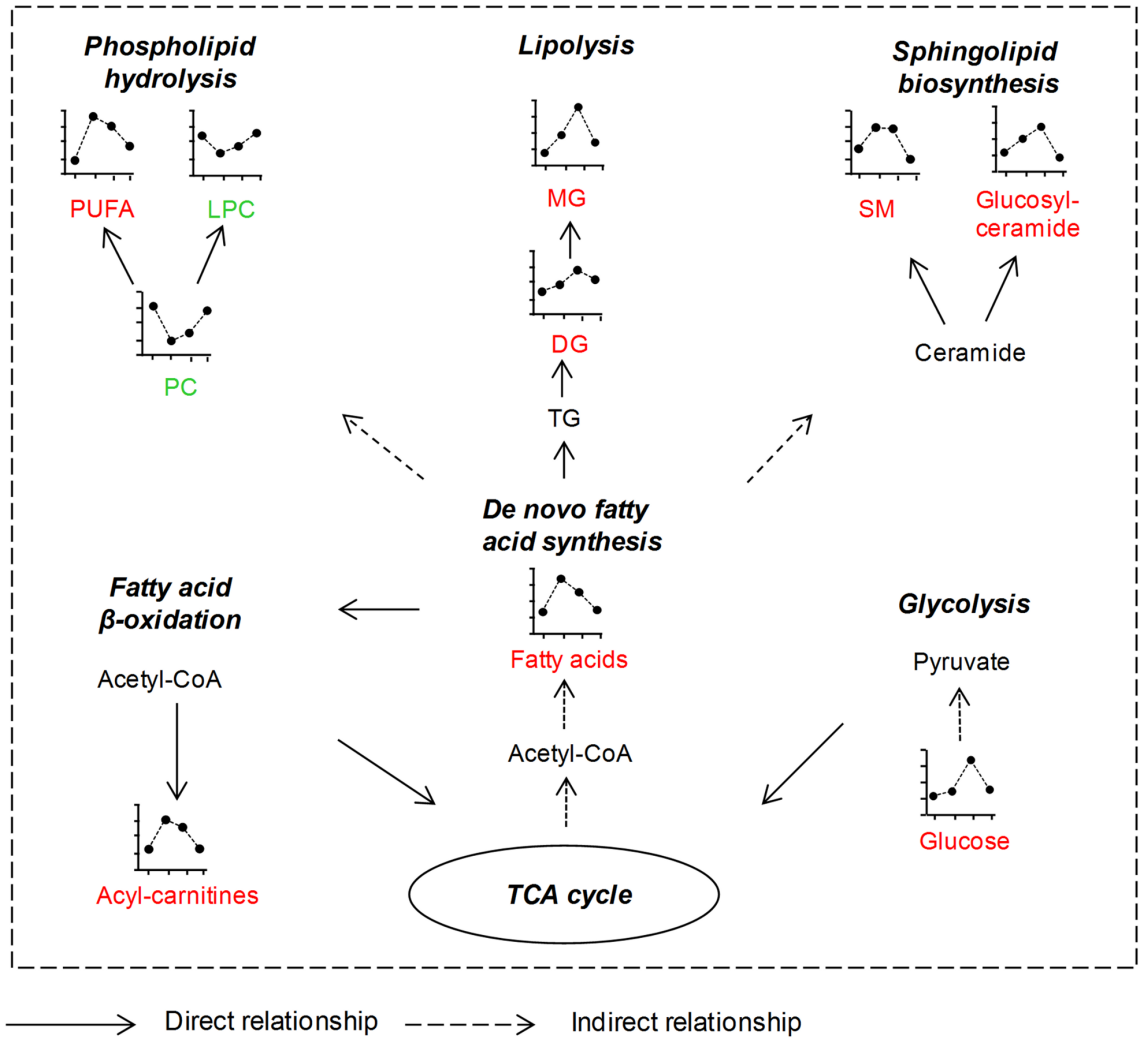
Major altered lipid and energy metabolic pathways in dengue patients. The four points on each line represent the levels of each metabolite at control, Visit 1, Visit 2 and Visit 3, respectively. Differential metabolites colored in red or green represent elevated or decreased change trend at acute stages (within 1 week) after dengue virus infection compared with healthy control subjects. These changes are reversible and there is no significant difference for most of the differential metabolites between healthy controls and dengue patients at convalescence stage (week 3–4). Black-colored metabolites either indicate similar level in healthy controls and dengue fever patients or are undetectable in this study. PC, phosphatidylcholine; PUFA, polyunsaturated fatty acid; LPC, lysophosphatidylcholine; MG, monoacylglyceride; DG, diacylglyceride; TG, triacylglyceride; SM, sphingomyelin.

We used two well-known pathway analysis tools- MetaboAnalyst and IPA, to determine if the metabolites identified here revealed underlying biochemical pathways. Both these tools provided similar results, revealing that disturbed metabolic pathways included fatty acid biosynthesis, fatty acid *β*-oxidation, phospholipid catabolism, arachidonic acid metabolism, sphingolipid metabolism, tryptophan metabolism, steroid hormone biosynthesis, purine metabolism, heme degradation pathway, bile acid biosynthesis, etc (**Figures S4**). The Networks function in IPA indicated that the most significant changes happened with lipid metabolism and energy production pathways, which is summarized in **Figure 4**.

### Global serum lipid changes with dengue fever

**Figure 5:**
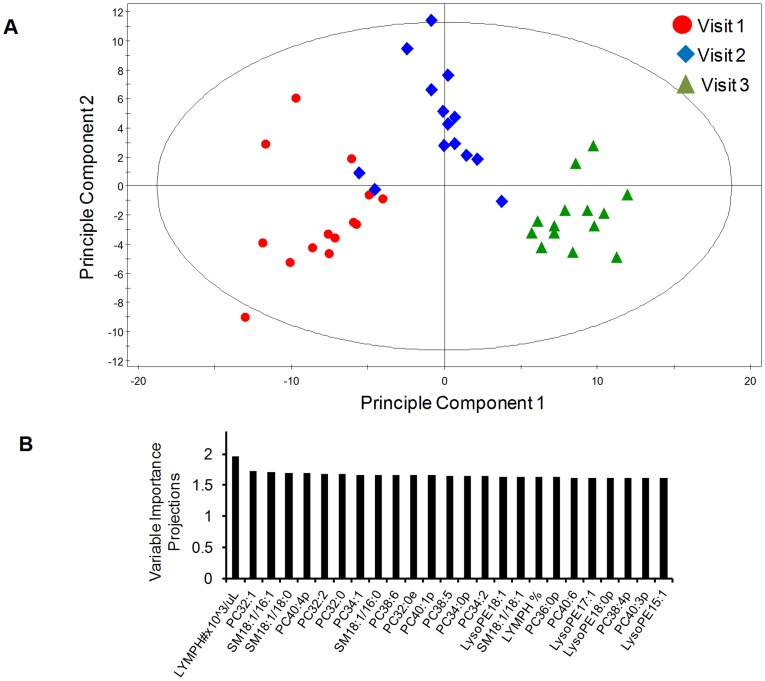
Serum lipids and blood chemistry describes distinct time points in primary dengue infection. **A.** Orthogonal partial least-squares discriminant analysis (OPLS-DA) models of median-centred and standard deviation scaled glycerophospholipids and sphingolipids and blood chemistry parameters collected from Visit 1 (1 to 3 days after the onset of fever; febrile phase), Visit 2 (4 to 7 days post fever onset; defervescence phase) and Visit 3 (21 to 27 days post fever onset; convalescence phase). **B.** OPLS-DA VIP plot showing the association of serum glycerophospholipids and sphingolipids with the blood chemistry parameters. Only parameters of VIP values >1.5 are shown. LYMPH#x10∧3/uL, lymphocyte numbers; LYMPH %, percentage of lymphocytes.

**Figure 6:**
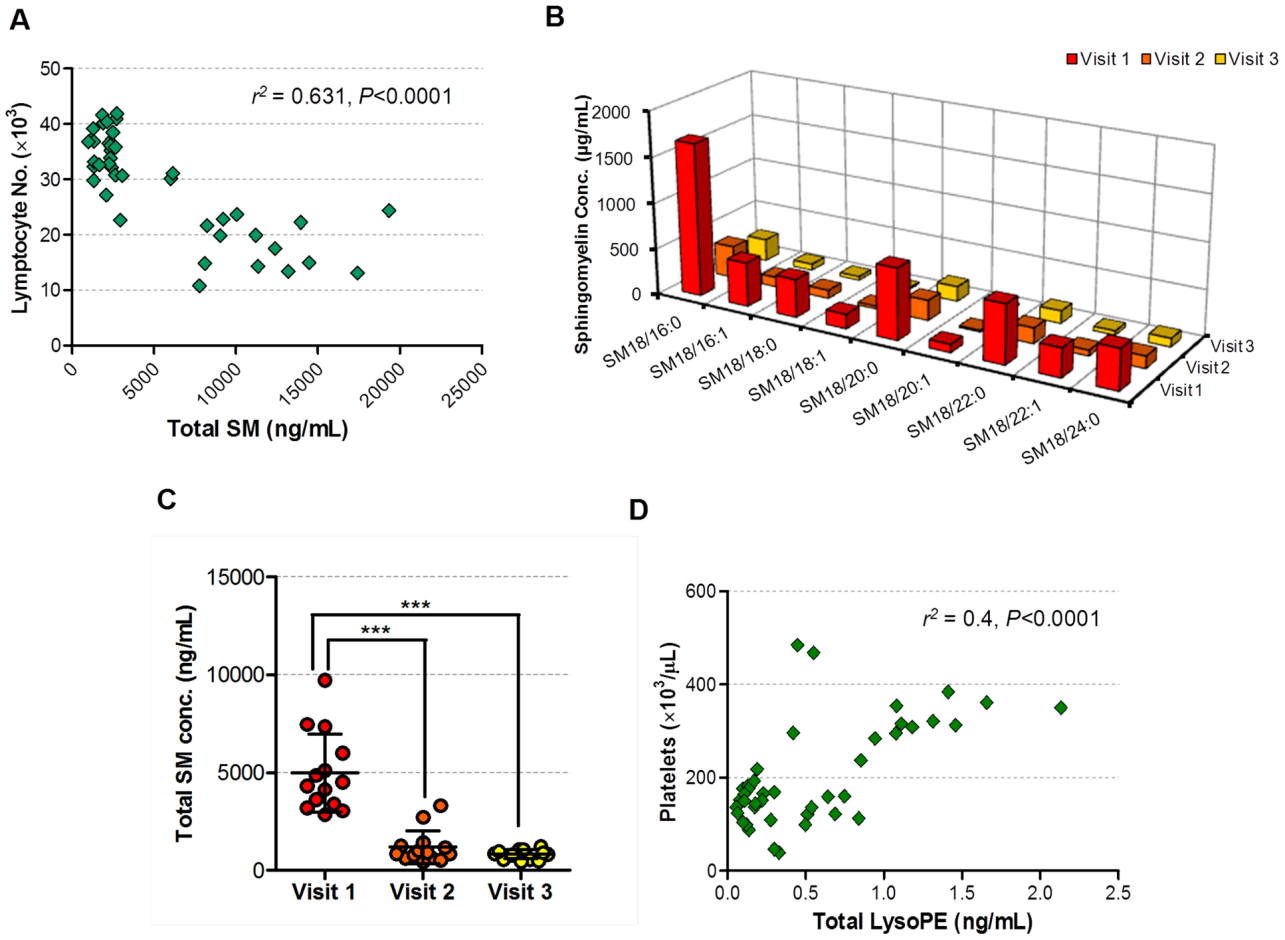
Correlation of serum lipids with specific serological parameters. **A.** Linear correlation of lymphocyte numbers with total sphingomyelin (SM). **B.** Quantitative mass spectrometry analysis of individual SM species. **C.** Scatter plot showing the temporal trend of total SM during dengue fever. *** *P*<0.0001. **D.** Linear correlation of platelet numbers with total lysoPE.

OPLS-DA revealed distinct temporal serum lipidome profiles with DENV infection (**Figure 5A**). To understand the lipids and blood chemistry parameters responsible for the differentiation of the three visits in the OPLS scores plot, the VIP plot was generated (**Figure 5B**). Lymphocyte numbers, SMs, PCs and lysoPEs influenced the model most significantly. We noted strong negative correlation between total SM with lymphocyte percentage (Pearson *r^2^* = 0.631, 95% C.I. = −0.8848 to −0.6467, *p*<0.0001; **Figures 6A** **, S5A**). The downward trend of SM levels across the visits (repeated measures 1-way ANOVA, *p*<0.0001) was dominated by SM d18:1/16:0 (*m/z* 703.7→184.1) which represented ∼1/3 of total SM and exhibited a 5-fold decrease from Visit 1 to 2 (**Figure 6B**). In cytotoxic T lymphocytes, acid sphingomyelinase, the enzyme that hydrolyzes SM to Cer, is a critical factor in the secretory granule-mediated cell death of virus infected cells [31]. Concordant with SM hydrolysis to ceramides [32], we observed increased ceramide levels paralleling the attenuation of SM (**Figure S5B**). Unlike the more biologically inert SMs, Cers are potent apoptosis-inducers, and their glycosylated forms, MHexCers or phosphorylated forms, C1Ps are bioactive sphingolipids [33]. The initial low Cer levels were not due to its glycosylation or phosphorylation which remained low (**Figures S5C, S5D**), but plausibly compartmentized as the less bioactive SM during the early onset of DF. This is evidenced by the relatively high SMs levels in the febrile phase compared to the later defervescence and convalescence phases (**Figure 6C**). The development of DENV-specific CD4+ and CD8+ T-lymphocyte responses [34], and the activation and contraction of antiviral CD8+ T lymphocytes in the defervescence and convalescence phases of dengue fever has previously been documented [35]. Therefore, the decrease in SM and increase in Cer correlated well with the action of T lymphocyte sphingomyelinase on SM and Cer levels in generating cytotoxic granules [31].

In our study cohort, MHexCers significantly increased from Visit 1 to Visit 3 (repeated measures 1-way ANOVA, *p*<0.01; **Figure S5C**), verifying our untargeted metabolomics results. Conversely, the higher glycosphingolipid, LacCer did not show significant differences across the visits (**Figure S5E**).

In our study we found many lysoPEs and pPEs closely correlated to platelet numbers (Pearson *r* ^2^ = 0.263–0.4, *p*<0.0001; **Figures 6C** **, S6A**). By contrast, PEs did not correlate with platelet numbers (Pearson *r* ^2^ = 0.0404, 95% C.I. = −0.4760 to 0.1096, *p* = 0.217). In addition, their temporal trends differed from pPEs and lysoPEs (**Figures S5B–D**). These pPEs are polyunsaturated and long-chained lipids (34p:2, 36p:4, 38p:4; 38p:5, 38p:6 and 40p:6. Plasmalogen glycerophospholipids are characterized by vinyl ether bonds at *sn*-1 position of the glycerol backbone, and two predominant glycerophospholipids are PEs and PCs. We also found that LPEs and pPEs strongly correlated to white blood cell count (Pearson *r* ^2^ = 0.425, *p*<0.0001). pPEs with no or single double bonds and of chain lengths more than 40 carbons correlated poorly, suggesting apparent alkyl chain length specificity to white blood cell count. Furthermore, univariate 1-way ANOVA showed that lysoPEs are significantly increased between Visits 2 and 3 (*p*<0.0001), and to a lesser degree between Visits 1 and 2 (*p*<0.05; **Figure S5B**). Taken together, our combined lipidome and blood chemistry analyses revealed that SMs are associated with early host response whereas lysoPEs are more associated with late recovery responses.

## Discussion

DF is a self-limiting febrile illness and in most DF cases, there is complete recovery with little to no manifestations of chronic negative clinical consequences, clearly indicating human-adapted protective mechanisms against DENV in DF patients. The protective mechanisms are complex and unfortunately, poorly studied, in part due to the focus on heterotypic infection and DHF/DSS. The study of the recovery of DF from a physiologic perspective is therefore important in advancing our understanding how humans mount a defense and recover from DENV infection and potentially other flaviviruses-induced illnesses. Our current integrated metabolomics and lipidomics study provides an extensive map of physiologic changes in adult DF patients, through which we have identified altered metabolic pathways linked to DENV infection. The relatively large population size (44 patients and 50 healthy controls) with repeated sampling at multiple time-points of infection, including early febrile, defervescence and convalescence stages, captured dynamic changes in metabolites and the discovery of reliable differential metabolites that were closely associated with dengue pathophysiology. By using both LC-MS/MS and GC-MS, we found that approximately 200 serum metabolites were significantly changed, of which 60 were identified. Lipids, which form a biochemically important subclass of metabolites, were also independently verified and studied. These metabolites constituted a mix of pro- as well as anti-inflammatory mediators, suggesting the rapid host resolution to limit excessive inflammation and curtail further exacerbation of DENV infection. Furthermore, these metabolites showed reversible trend changes, indicating that they might be able to potentially serve as the basis to develop prognostic markers of the disease.

Infection of humans with DENV causes early pronounced immunological reactions that shift towards pro-inflammatory responses [36]. While acute inflammatory response is necessary in initiating pathogen killing, activation of anti-inflammatory and pro-resolution processes is important to prevent excessive pathological inflammatory damage to the host. Omega-6 PUFAs, which are released from cell-membrane phospholipids by phospholipid hydrolysis, including AA and AA-derived mediators such as prostaglandins, thromboxanes, hydroxyeicosatetraenoic acids and leukotrienes (collectively known as eicosanoids) are potent mediators of inflammation and are involved in modulating both the intensity and duration of inflammatory responses [37], [38]. Contrary to the effects of AA, omega-3 PUFAs, e.g. DHA are known for their anti-inflammatory activity [39]. Our results showed an elevated trend of pro- and anti-inflammatory PUFAs during the febrile stage of DENV infection, including both AA and DHA, as are total hydroxyeicosatetraenoic acids [40]. Such effects of active resolution, where AA-associated inflammation incited early during DENV infection is concomitantly resolved by DHA-derived anti-inflammatory mediators, and the suppressed production of pro-inflammatory cytokines [39], is believed to embrace the current model of inflammation whereby the return to homeostasis is an active process, as opposed to a passive one [41]. We also found increased levels of cortisol, a major, cytokine-activated glucocorticoid of potent anti-inflammatory and immunosuppressive properties in the febrile stage of DF. Viral infections are capable of inducing an increase of cortisol in serum during DENV infection, it had been reported that cortisol level at early febrile stage was significantly higher than its level at convalescent stage [42], consistent with our results. The functional role of cortisol in restraining inflammatory and immune responses by inhibiting the production of cytokines and other pro-inflammatory mediators [43] could possibly down-regulate immune responses and protect the DF patients against cytokine-mediated pathologies. Consistent with elevated oxidative stress in adult DF patients [44] and elevated inosine concentrations in the extracellular space at times of inflammation [45], we found an increased serum inosine in the febrile phase. Inosine, potentially formed through nitrosative deamination of adenosine, has been shown to exert wide-ranging anti-inflammatory effects both *in vivo* and *in vitro*, such as inhibition of pro-inflammatory cytokine/chemokine production and the enhancement of anti-inflammatory cytokine IL-10 production [46]. Taken together, our metabolomics results reveal a dynamic metabolite flux in dengue where an acute inflammation is limited by swift, dampening responses to restore homeostasis.

In parallel, host immunity has interested dengue investigators in exploring the mechanisms in the immune system underlying DF pathogenesis. In primary infections, neutralizing antibody responses develop after DENV infection and provide persistent protection against the same serotype even in repeated infections; even though this may lead to the enhancement of disease severity upon challenges of a following heterotypic DENV infection. Increasing evidence demonstrate that self-antigenic glycerophospholipids or glycosphingolipids activate the innate immune system, noticeably NKT cells in microbial infections [47]–[50]. Two MHexCers, GlcCer and GalCer, ceramides with β-linked glucose and α-linked galactose, produced by sphingolipid biosynthesis, have been implicated in invariant natural killer T-cell (*i*NKT) activation during microbial infections [47], [49], [50], and also NKT cells, which have been implicated in DENV clearance in DF patients [51], [52]. Consistent with this, we found increased GlcCer levels with the visits and followed the same trend as lymphocyte numbers. Other immune cells such as dendritic cells, CD4+ and CD8+ lymphocytes play major roles in viral clearance and as targets of DENV infection. On the basis of the aforementioned observations, we postulated that the relationship between SM, Cer concentrations and immune cells describe multiple early, molecular roles of sphingolipids in mediating the host interactions during DF. Central among the sphingolipid enzymes is acid sphingomyelinase, which has been implicated in numerous immune responses including, T lymphocyte activation and positive infection by ebola viruses [53], entry of measles viruses into dendritic cells [54] and exocytosis of cytolytic granules by T cells [31]. The binding of extracellular ceramide to the leukocyte mono-immunoglobulin-like receptor 3 has been recently reported in repressing mast cell activation [55] and serum ceramides could potentially influence mast cell surveillance in DENV infection [56]. Likewise, we also observed the correlation of LPEs and pPEs with white blood cells and may potentially be related to *i*NKT activation. Plasmalogen lysoPEs (pLPE) are self-antigenic and are required for *i*NKT stimulation under both physiological conditions and when challenged with bacteria [48]. We expect that, our findings that sphingolipids are associated with lymphocytes during DF may further unravel the significance of T-cell responses and warrants further investigations. The lack of any significant change in phosphatidic acids and its lyso form was intriguing given its commonly assigned roles in platelet activation. However, it could be because unlike DHF, DF does not entail severe thrombocytopenia and our blood chemistry assays suggest the same.

In the wide spectrum of responses occurring simultaneously with pathogenic invasions, some metabolic changes may appear to be general and others more specific. In influenza 2009 pandemic H1N1 infection, extensive changes in serum eicosanoid (e.g. prostaglandin-F1α, prostaglandin-G, LTA_4_) and linolenic acid levels were observed [57]. While linolenic acid is a FFA common to both H1N1 infection and dengue infection, there was no observable difference in eicosanoids in DF. Another plausible distinctive metabolome change in DF compared to H1N1 is the xanthinine derivatives. In 2009 pandemic H1N1 infection, serum methylxanthine levels were lower during infection relative to after treatment [57]. This result can be interpreted in two ways: theophylline, a methylxanthine has been shown to elicit immunomodulator, anti-inflammatory, and bronchoprotective properties [58] and such properties may help attenuate the bronchi damaging effects of 2009 pandemic H1N1 virus as *i*) part of the host response [59] or *ii*) constitute part of the unspecified treatment. Regardless of which is the reason, methylxanthine points towards the lung-injury specific consequence of 2009 pandemic H1N1 infection. On the other hand, in DF, circulating inosine and hypoxanthine levels increased in the febrile phase where systemic inflammation may be concerned [60], [61]. While studies of increased inosine and hypoxanthine levels were demonstrated in models of chronic systemic inflammation, it is conceivable that similar events could be observed in infectious diseases where acute bouts of inflammation occur. During inflammation, increased adenosine-to-inosine editing in cytokine mRNA may interfere with the degradation of cytokine transcripts and support the early pro-inflammatory and resolving responses necessary in the early phase of dengue. This may be reflected in a mixture of pro- and anti-inflammatory cytokines such as significant IL-4, IL-8 and IFNγ increases in the febrile phase of DF [16].

On the other hand, certain metabolite changes may be more general across different pathogenic infections. For example, we found increased phenylalanine levels in the early stages of DF. Tetrahydrobiopterin (BH4) is a co-factor for phenylalanine (4)-hydroxylase (PHA), an enzyme required for converting phenylalanine to tyrosine, and also NO synthase (NOS) [62]. Through immune and inflammatory responses-mediated up-regulation of NOS, NO is elevated during dengue infection [63], [64]. Therefore, NOS competes with PHA for BH4 and this causes an accumulation of unconverted phenylalanine in the blood. This is aggravated in the elevated oxidative stress status in DF patients [44] where superoxide and peroxynitrite increases BH4 oxidation, thereby depleting the available BH4 pool. Yet, an increased level of phenylalanine has been commonly observed in other infectious diseases [65] and given NO's diverse functions in immunity and inflammation responses it is not surprising that such changes could occur in both bacterial and viral infections [66], [67]. Another general effect is energy metabolism. In this study we identified ten acylcarnitines, which are essential intermediates of fatty acid *β*-oxidation (FAO). FAO in liver is suppressed during infection and inflammation, and increased FFA substrates are then moved away from oxidation and directed toward re-esterification into TG [68]. The elevated levels of acylcarnitines suggest disturbed FAO in DF patients, which is in agreement with the observation that the TCA cycle was affected in an endothelial cell line infected with DENV [14]. Proteomics and lipidomics work by Diamond *et al.*, showed a shift in energy metabolism during HCV infection in Huh7.5 cells, plausibly as a compensatory measure for the energy-demanding biosynthesis [15]. Similarly, a metabolomics study on severe pneumonia identified energy metabolism as a perturbed pathway [10]. Our relatively large number of human samples not only corrobated the *in vitro* findings but also showed that energy metabolism is likely to be a general perturbed pathway in acute infections. In the absence of large datasets of host-pathogen ‘functional omic’ datasets, we are limited in our ability to make comparisons and evaluate pathways specific to either bacterial or viral infections in humans. However, with increasing need to comprehensively capture the complexity of host-pathogen interactions, and aided by advancements in mass spectrometry and computational biology [69], we expect more datasets made available to the community for meaningful comparisons.

The limitations of our study can be discussed in the context of the host. Firstly, since the current study is restricted to adult primary infections the findings still need to be expanded and evaluated in pediatric patients as well as patients with secondary infections. Secondly, DENV progressively infects a multitude of cells and organs, including dendritic cells, monocytes, macrophages, endothelial cells and liver [70]–[72]. When we approached the host response from a systemic perspective, changes of the chemical composition in the serum could reflect tissue lesions, organ dysfunctions, pathological states and also compensatory responses. Thus pinpointing the source or cause of these changes can be challenging. Nevertheless, our results strongly raise the implication of distinctive metabolome changes with specific physiological responses at different phases of dengue infection. It will require increasing volumes of investigative work to tease apart the sources of these metabolites, including lipids. More importantly, as the body of metabolomic work on *in vivo* viral infections grows, the potential to derive distinctive and accurate biomarkers alluding to host responses of various viral infections increases.

In summary, our findings provide a first detailed description of the metabolome changes in patients with acute dengue infection and offer a global molecular view that leads to the overall homeostatic physiological outcome of DF. In addition to providing host-pathogen insights into the underlying mechanism in symptom manifestation, metabolites identified in this study might be used both to monitor disease progression as well as evaluate the efficacy of therapeutic interventions. Furthermore, certain pathways might serve as useful therapeutic targets to alleviate severity of dengue. Such work would be our next endeavour.

## Supporting Information

Figure S1 **Workflow of serum metabolomics study based on LC-MS/MS and GC-MS analysis.**
(TIF)

Figure S2 **Schematic workflow of mass spectrometry-based lipidomics on serum samples collected from EDEN study.**
(TIF)

Figure S3 **Identification of hexanoylcarnitine as a differential metabolite in dengue fever.** **A.** the extracted ion chromatogram (EIC) and matched formula of the ion *m/z* 260.18. **B.** MS/MS spectrum and proposed fragmentation pathways of the ion *m/z* 260.18. **C.** MS/MS spectrum of a commercial standard hexanoylcarnitine.
(TIF)

Figure S4 **Pathway analysis results by MetaboAnalyst.**
(TIF)

Figure S5 **Temporal trends of A. lymphocyte numbers and B. Cer, C. GlcCer, D. C1P and E. LacCer levels.** *p<0.05; **p<0.01.
(TIF)

Figure S6 **Temporal trends of A. platelet numbers and B. lysoPE, C. pPE and D. PE levels.** *p<0.05; ***p<0.0001.
(TIF)

Figure S7 **Scatter plots for the major differential metabolite classes.** **A.** arachidonic acid **B.** hexanoylcarnitine **C.** glucosylceramide **D.** cortisol **E.** inosine **F.** biliverdine **G.** PC 38∶8 **H.** tryptophan **I.** phenylalanine. The black band near the middle of the plot is the mean peak area of the metabolite.
(TIF)

Table S1 **Reproducibility evaluation based on selected peaks in quality control samples.**
(DOCX)

Table S2 **Optimized lipid-dependent mass spectrometry parameters for MRM.**
(XLSX)

Table S3 **Linearity, Limit of Detection (LOD) and Limit of Quantitation (LOQ) of sphingolipids. LOD and LOQ were determined according to the International Conference on Harmonization (ICH) guidelines.**
(XLSX)

Table S4 **Identified differential metabolites from LC-MS/MS analysis.**
(DOCX)

Table S5 **Unidentified differential metabolites from LC-MS/MS analysis.**
(DOCX)

## References

[1] Halstead SB, Suaya JA, Shepard DS (2007) The burden of dengue infection. Lancet 369: 1410–1411. 17467495 10.1016/S0140-6736(07)60645-X

[2] Gubler DJ (2002) The global emergence/resurgence of arboviral diseases as public health problems. Arch Med Res 33: 330–342. 12234522 10.1016/s0188-4409(02)00378-8

[3] Guzman MG, Halstead SB, Artsob H, Buchy P, Farrar J, et al (2010) Dengue: a continuing global threat. Nat Rev Microbiol 8: S7–16. 21079655 10.1038/nrmicro2460 PMC4333201

[4] Gubler DJ (1998) Dengue and dengue hemorrhagic fever. Clin Microbiol Rev 11: 480–496. 9665979 10.1128/cmr.11.3.480 PMC88892

[5] Nicholson JK, Lindon JC, Holmes E (1999) ‘Metabonomics’: understanding the metabolic responses of living systems to pathophysiological stimuli via multivariate statistical analysis of biological NMR spectroscopic data. Xenobiotica 29: 1181–1189. 10598751 10.1080/004982599238047

[6] Sreekumar A, Poisson LM, Rajendiran TM, Khan AP, Cao Q, et al (2009) Metabolomic profiles delineate potential role for sarcosine in prostate cancer progression. Nature 457: 910–914. 19212411 10.1038/nature07762 PMC2724746

[7] Wang Z, Klipfell E, Bennett BJ, Koeth R, Levison BS, et al (2011) Gut flora metabolism of phosphatidylcholine promotes cardiovascular disease. Nature 472: 57–63. 21475195 10.1038/nature09922 PMC3086762

[8] Vinayavekhin N, Homan EA, A S (2010) Exploring disease through metabolomics. ACS Chem Biol 15: 91–103. 10.1021/cb900271r 20020774

[9] Al-Mubarak R, Vander Heiden J, Broeckling CD, Balagon M, Brennan PJ, et al (2011) Serum metabolomics reveals higher levels of polyunsaturated fatty acids in lepromatous leprosy: potential markers for susceptibility and pathogenesis. PLoS Negl Trop Dis 5: e1303. 21909445 10.1371/journal.pntd.0001303 PMC3167790

[10] Laiakis EC, Morris GA, Fornace AJ, Howie SR (2010) Metabolomic analysis in severe childhood pneumonia in the Gambia, West Africa: findings from a pilot study. PLoS One 5: e12655. 20844590 10.1371/journal.pone.0012655 PMC2936566

[11] Heaton NS, Perera R, Berger KL, Khadka S, Lacount DJ, et al (2010) Dengue virus nonstructural protein 3 redistributes fatty acid synthase to sites of viral replication and increases cellular fatty acid synthesis. Proc Natl Acad Sci U S A 107: 17345–17350. 20855599 10.1073/pnas.1010811107 PMC2951450

[12] Rothwell C, Lebreton A, Young Ng C, Lim JY, Liu W, et al (2009) Cholesterol biosynthesis modulation regulates dengue viral replication. Virology 389: 8–19. 19419745 10.1016/j.virol.2009.03.025

[13] Zaitseva E, Yang ST, Melikov K, Pourmal S, Chernomordik LV (2010) Dengue virus ensures its fusion in late endosomes using compartment-specific lipids. PLoS Pathog 6: e1001131. 20949067 10.1371/journal.ppat.1001131 PMC2951369

[14] Birungi G, Chen SM, Loy BP, Ng ML, Li SF (2010) Metabolomics approach for investigation of effects of dengue virus infection using the EA.hy926 cell line. J Proteome Res 9: 6523–6534. 20954703 10.1021/pr100727m

[15] Diamond DL, Syder AJ, Jacobs JM, Sorensen CM, Walters KA, et al (2010) Temporal proteome and lipidome profiles reveal hepatitis C virus-associated reprogramming of hepatocellular metabolism and bioenergetics. PLoS Pathog 6: e1000719. 20062526 10.1371/journal.ppat.1000719 PMC2796172

[16] Kumar Y, Liang C, Bo Z, Rajapakse JC, Ooi EE, et al (2012) Serum Proteome and Cytokine Analysis in a Longitudinal Cohort of Adults with Primary Dengue Infection Reveals Predictive Markers of DHF. PLoS Negl Trop Dis 6: e1887. 23209847 10.1371/journal.pntd.0001887 PMC3510095

[17] Low JG, Ooi EE, Tolfvenstam T, Leo YS, Hibberd ML, et al (2006) Early Dengue infection and outcome study (EDEN) - study design and preliminary findings. Ann Acad Med Singapore 35: 783–789. 17160194

[18] Low JG, Ong A, Tan LK, Chaterji S, Chow A, et al (2011) The early clinical features of dengue in adults: challenges for early clinical diagnosis. PLoS Negl Trop Dis 5: e1191. 21655307 10.1371/journal.pntd.0001191 PMC3104968

[19] WHO (1997) Dengue Hemorrhagic Fever: diagnosis, treatment, prevention, and control. Geneva: World Health Organization.

[20] Bligh EG, Dyer WJ (1959) A rapid method of total lipid extraction and purification. Can J Biochem Physiol 37: 911–917. 13671378 10.1139/o59-099

[21] Ng DP, Salim A, Liu Y, Zou L, Xu FG, et al (2012) A metabolomic study of low estimated GFR in non-proteinuric type 2 diabetes mellitus. Diabetologia 55: 499–508. 22038517 10.1007/s00125-011-2339-6

[22] Han X, Gross RW (2005) Shotgun lipidomics: electrospray ionization mass spectrometric analysis and quantitation of cellular lipidomes directly from crude extracts of biological samples. Mass Spectrom Rev 24: 367–412. 15389848 10.1002/mas.20023

[23] Shaner RL, Allegood JC, Park H, Wang E, Kelly S, et al (2009) Quantitative analysis of sphingolipids for lipidomics using triple quadrupole and quadrupole linear ion trap mass spectrometers. J Lipid Res 50: 1692–1707. 19036716 10.1194/jlr.D800051-JLR200 PMC2724058

[24] Brugger B, Erben G, Sandhoff R, Wieland FT, Lehmann WD (1997) Quantitative analysis of biological membrane lipids at the low picomole level by nano-electrospray ionization tandem mass spectrometry. Proc Natl Acad Sci U S A 94: 2339–2344. 9122196 10.1073/pnas.94.6.2339 PMC20089

[25] Pluskal T, Castillo S, Villar-Briones A, Oresic M (2010) MZmine 2: modular framework for processing, visualizing, and analyzing mass spectrometry-based molecular profile data. BMC Bioinformatics 11: 395. 20650010 10.1186/1471-2105-11-395 PMC2918584

[26] Chen J, Zhao X, Fritsche J, Yin P, Schmitt-Kopplin P, et al (2008) Practical approach for the identification and isomer elucidation of biomarkers detected in a metabonomic study for the discovery of individuals at risk for diabetes by integrating the chromatographic and mass spectrometric information. Anal Chem 80: 1280–1289. 18193893 10.1021/ac702089h

[27] Zuniga A, Li L (2011) Ultra-high performance liquid chromatography tandem mass spectrometry for comprehensive analysis of urinary acylcarnitines. Anal Chim Acta 689: 77–84. 21338760 10.1016/j.aca.2011.01.018

[28] Xia J, Psychogios N, Young N, Wishart DS (2009) MetaboAnalyst: a web server for metabolomic data analysis and interpretation. Nucleic Acids Res 37: W652–660. 19429898 10.1093/nar/gkp356 PMC2703878

[29] Nevalainen TJ, Losacker W (1997) Serum phospholipase A2 in dengue. J Infect 35: 251–252. 9459397 10.1016/s0163-4453(97)92966-2

[30] Klassen P, Furst P, Schulz C, Mazariegos M, Solomons NW (2001) Plasma free amino acid concentrations in healthy Guatemalan adults and in patients with classic dengue. Am J Clin Nutr 73: 647–652. 11237944 10.1093/ajcn/73.3.647

[31] Herz J, Pardo J, Kashkar H, Schramm M, Kuzmenkina E, et al (2009) Acid sphingomyelinase is a key regulator of cytotoxic granule secretion by primary T lymphocytes. Nat Immunol 10: 761–768. 19525969 10.1038/ni.1757

[32] Nixon GF (2009) Sphingolipids in inflammation: pathological implications and potential therapeutic targets. Br J Pharmacol 158: 982–993. 19563535 10.1111/j.1476-5381.2009.00281.x PMC2785521

[33] Hannun YA (2000) Luberto (2000) Ceramide in the eukaryotic stress response. Trends Cell Biol 10: 73–80. 10652518 10.1016/s0962-8924(99)01694-3

[34] Gagnon SJ, Zeng W, Kurane I, Ennis FA (1996) Identification of two epitopes on the dengue 4 virus capsid protein recognized by a serotype-specific and a panel of serotype-cross-reactive human CD4+ cytotoxic T-lymphocyte clones. J Virol 70: 141–147. 8523518 10.1128/jvi.70.1.141-147.1996 PMC189798

[35] Sandalova E, Laccabue D, Boni C, Tan AT, Fink K, et al (2010) Contribution of herpesvirus specific CD8 T cells to anti-viral T cell response in humans. PLoS Pathog 6: e1001051. 20808900 10.1371/journal.ppat.1001051 PMC2924358

[36] Mathew A, Rothman AL (2008) Understanding the contribution of cellular immunity to dengue disease pathogenesis. Immunol Rev 225: 300–313. 18837790 10.1111/j.1600-065X.2008.00678.x

[37] Lewis RA, Austen KF, Soberman RJ (1990) Leukotrienes and other products of the 5-lipoxygenase pathway. Biochemistry and relation to pathobiology in human diseases. N Engl J Med 323: 645–655. 2166915 10.1056/NEJM199009063231006

[38] Serhan CN (2011) The resolution of inflammation: the devil in the flask and in the details. Faseb J 25: 1441–1448. 21532053 10.1096/fj.11-0502ufm PMC3228345

[39] Calder PC (2001) Polyunsaturated fatty acids, inflammation, and immunity. Lipids 36: 1007–1024. 11724453 10.1007/s11745-001-0812-7

[40] Seet RC, Lee CY, Lim EC, Quek AM, Yeo LL, et al (2009) Oxidative damage in dengue fever. Free Radic Biol Med 47: 375–380. 19427377 10.1016/j.freeradbiomed.2009.04.035

[41] Serhan CN, Brain SD, Buckley CD, Gilroy DW, Haslett C, et al (2007) Resolution of inflammation: state of the art, definitions and terms. Faseb J 21: 325–332. 17267386 10.1096/fj.06-7227rev PMC3119634

[42] Myo K, Soe T, Thein Thein M, Than Nu S, Tin Tin S, et al (1995) Serum cortisol levels in children with dengue haemorrhagic fever. J Trop Pediatr 41: 295–297. 8531262 10.1093/tropej/41.5.295

[43] Ruzek MC, Pearce BD, Miller AH, Biron CA (1999) Endogenous glucocorticoids protect against cytokine-mediated lethality during viral infection. J Immunol 162: 3527–3533. 10092810

[44] Gil L, Martinez G, Tapanes R, Castro O, Gonzalez D, et al (2004) Oxidative stress in adult dengue patients. Am J Trop Med Hyg 71: 652–657. 15569800

[45] Hellewell PG, Pearson JD (1983) Metabolism of circulating adenosine by the porcine isolated perfused lung. Circ Res 53: 1–7. 6861292 10.1161/01.res.53.1.1

[46] Hasko G, Kuhel DG, Nemeth ZH, Mabley JG, Stachlewitz RF, et al (2000) Inosine inhibits inflammatory cytokine production by a posttranscriptional mechanism and protects against endotoxin-induced shock. J Immunol 164: 1013–1019. 10623851 10.4049/jimmunol.164.2.1013

[47] Brennan PJ, Tatituri RV, Brigl M, Kim EY, Tuli A, et al (2011) Invariant natural killer T cells recognize lipid self antigen induced by microbial danger signals. Nat Immunol 12: 1202–1211. 22037601 10.1038/ni.2143 PMC3242449

[48] Facciotti F, Ramanjaneyulu GS, Lepore M, Sansano S, Cavallari M, et al (2012) Peroxisome-derived lipids are self antigens that stimulate invariant natural killer T cells in the thymus. Nat Immunol 13: 474–480. 22426352 10.1038/ni.2245

[49] Kawano T, Cui J, Koezuka Y, Toura I, Kaneko Y, et al (1997) CD1d-restricted and TCR-mediated activation of valpha14 NKT cells by glycosylceramides. Science 278: 1626–1629. 9374463 10.1126/science.278.5343.1626

[50] Mattner J, Debord KL, Ismail N, Goff RD, Cantu C 3rd, et al (2005) Exogenous and endogenous glycolipid antigens activate NKT cells during microbial infections. Nature 434: 525–529. 15791258 10.1038/nature03408

[51] Azeredo EL, De Oliveira-Pinto LM, Zagne SM, Cerqueira DI, Nogueira RM, et al (2006) NK cells, displaying early activation, cytotoxicity and adhesion molecules, are associated with mild dengue disease. Clin Exp Immunol 143: 345–356. 16412060 10.1111/j.1365-2249.2006.02996.x PMC1809585

[52] Zigmond E, Preston S, Pappo O, Lalazar G, Margalit M, et al (2007) Beta-glucosylceramide: a novel method for enhancement of natural killer T lymphoycte plasticity in murine models of immune-mediated disorders. Gut 56: 82–89. 17172586 10.1136/gut.2006.095497 PMC1856679

[53] Miller ME, Adhikary S, Kolokoltsov AA, Davey RA (2012) Ebolavirus requires acid sphingomyelinase activity and plasma membrane sphingomyelin for infection. J Virol 86: 7473–7483. 22573858 10.1128/JVI.00136-12 PMC3416309

[54] Avota E, Gulbins E, Schneider-Schaulies S (2011) DC-SIGN mediated sphingomyelinase-activation and ceramide generation is essential for enhancement of viral uptake in dendritic cells. PLoS Pathog 7: e1001290. 21379338 10.1371/journal.ppat.1001290 PMC3040670

[55] Izawa K, Yamanishi Y, Maehara A, Takahashi M, Isobe M, et al (2012) The Receptor LMIR3 Negatively Regulates Mast Cell Activation and Allergic Responses by Binding to Extracellular Ceramide. Immunity 37: 827–839. 23123064 10.1016/j.immuni.2012.08.018

[56] St John AL, Rathore AP, Yap H, Ng ML, Metcalfe DD, et al (2011) Immune surveillance by mast cells during dengue infection promotes natural killer (NK) and NKT-cell recruitment and viral clearance. Proc Natl Acad Sci U S A 108: 9190–9195. 21576486 10.1073/pnas.1105079108 PMC3107258

[57] Lu C, Jiang Z, Fan X, Liao G, Li S, et al (2012) A metabonomic approach to the effect evaluation of treatment in patients infected with influenza A (H1N1). Talanta 15;100: 51–56. 10.1016/j.talanta.2012.07.076 23141311

[58] Tilley SL (2011) Methylxanthines in asthma. Handb Exp Pharmacol 200: 439–456. 10.1007/978-3-642-13443-2_17 20859807

[59] Guarner J, Falcón-Escobedo R (2009) Comparison of the pathology caused by H1N1, H5N1, and H3N2 influenza viruses. Arch Med Res 40: 655–661. 20304252 10.1016/j.arcmed.2009.10.001

[60] Yang JH, Luo X, Nie Y, Su Y, Zhao Q (2003) Widespread inosine-containing mRNA in lymphocytes regulated by ADAR1 in response to inflammation. Immunology 109: 15–23. 12709013 10.1046/j.1365-2567.2003.01598.x PMC1782949

[61] Mangerich A, Knutson CG, Parry NM, Muthupalani S, Ye W, et al (2012) Infection -induced colitis in mice causes dynamic and tissue-specific changes in stress response and DNA damage leading to colon cancer. Proc Natl Acad Sci USA 109: E1820–1829. 22689960 10.1073/pnas.1207829109 PMC3390855

[62] Shi W, Meininger CJ, Haynes TE, Hatakeyama K, Wu G (2004) Regulation of tetrahydrobiopterin synthesis and bioavailability in endothelial cells. Cell Biochem Biophys 41: 415–434. 15509890 10.1385/CBB:41:3:415

[63] Neves-Souza PC, Azeredo EL, Zagne SM, Valls-de-Souza R, Reis SR, et al (2005) Inducible nitric oxide synthase (iNOS) expression in monocytes during acute Dengue Fever in patients and during in vitro infection. BMC Infect Dis 5: 64. 16109165 10.1186/1471-2334-5-64 PMC1208887

[64] Takhampunya R, Padmanabhan R, Ubol S (2006) Antiviral action of nitric oxide on dengue virus type 2 replication. J Gen Virol 87: 3003–3011. 16963759 10.1099/vir.0.81880-0

[65] Wannemacher RW Jr, Klainer AS, Dinterman RE, Beisel WR (1976) The significance and mechanism of an increased serum phenylalanine-tyrosine ratio during infection. Am J Clin Nutr 29: 997–1006. 822705 10.1093/ajcn/29.9.997

[66] Nathan CF, Hibbs JB Jr (1991) Role of nitric oxide synthesis in macrophage antimicrobial activity. Curr Opin Immunol 3: 65–70. 1711326 10.1016/0952-7915(91)90079-g

[67] Akaike T, Maeda H (2000) Nitric oxide and virus infection. Immunology 101: 300–308. 11106932 10.1046/j.1365-2567.2000.00142.x PMC2327086

[68] Suvarna JC, Rane PP (2009) Serum lipid profile: a predictor of clinical outcome in dengue infection. Trop Med Int Health 14: 576–585. 19309479 10.1111/j.1365-3156.2009.02261.x

[69] Goh WW, Lee YH, Chung M, Wong L (2012) How advancement in biological network analysis methods empowers proteomics. Proteomics 12: 550–563. 22247042 10.1002/pmic.201100321

[70] Jessie K, Fong MY, Devi S, Lam SK, Wong KT (2004) Localization of dengue virus in naturally infected human tissues, by immunohistochemistry and in situ hybridization. J Infect Dis 189: 1411–1418. 15073678 10.1086/383043

[71] Noisakran S, Onlamoon N, Songprakhon P, Hsiao HM, Chokephaibulkit K, et al (2010) Cells in dengue virus infection in vivo. Adv Virol 2010: 164878. 22331984 10.1155/2010/164878 PMC3276057

[72] Murphy BR, Whitehead SS (2011) Immune response to dengue virus and prospects for a vaccine. Annu Rev Immunol 29: 587–619. 21219187 10.1146/annurev-immunol-031210-101315

