# Supplementary figures and images for: Serum Metabolome and Lipidome Changes in Adult Patients with Primary Dengue Infection
Source: PLoS Negl Trop Dis. 2013 Aug 15;7(8):e2373. doi: 10.1371/journal.pntd.0002373 (PMC3744433; doi:10.1371/journal.pntd.0002373)

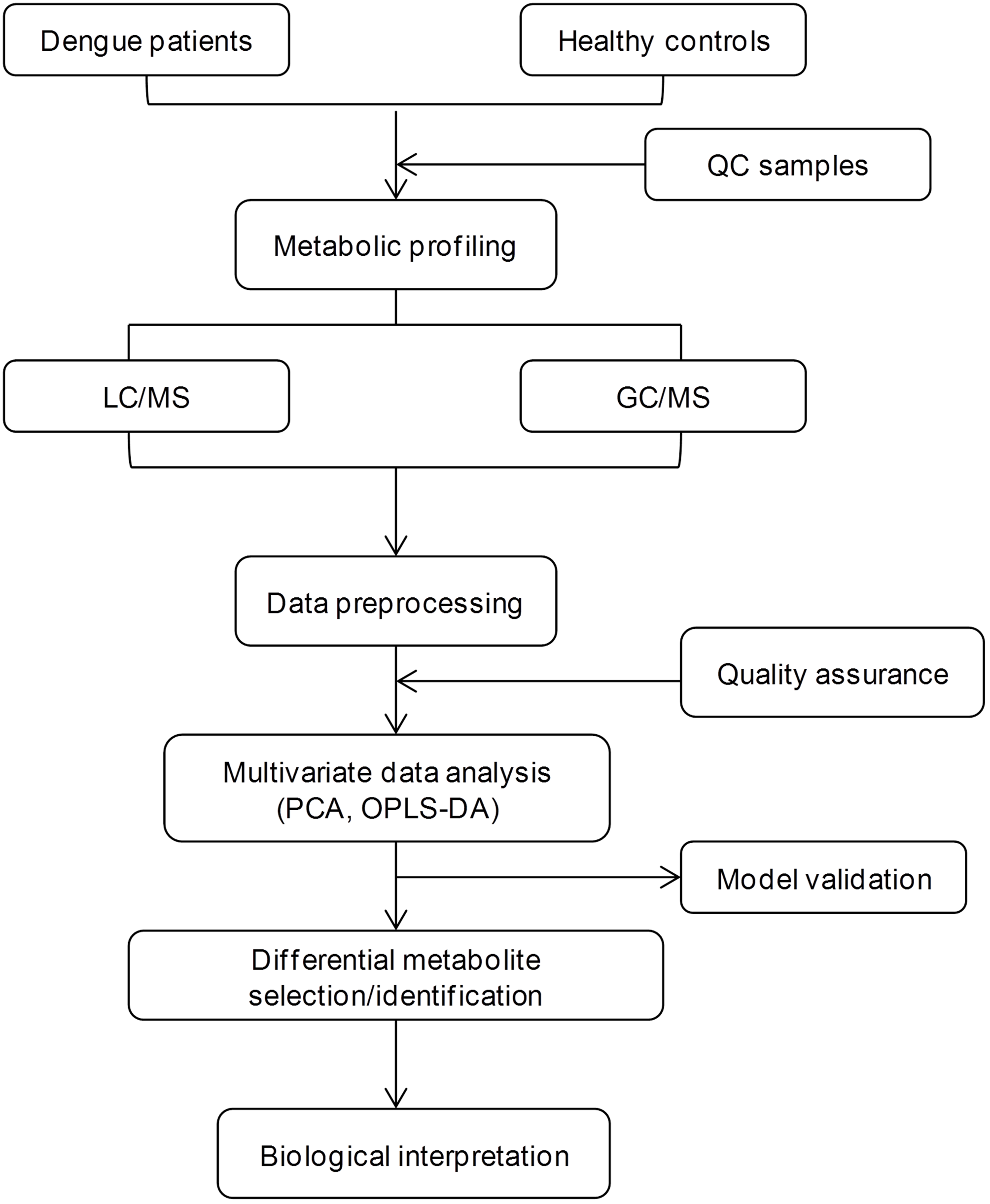

Supplement: Figure S1 — Workflow of serum metabolomics study based on LC-MS/MS and GC-MS analysis. (TIF) [file pntd.0002373.s001.tif]

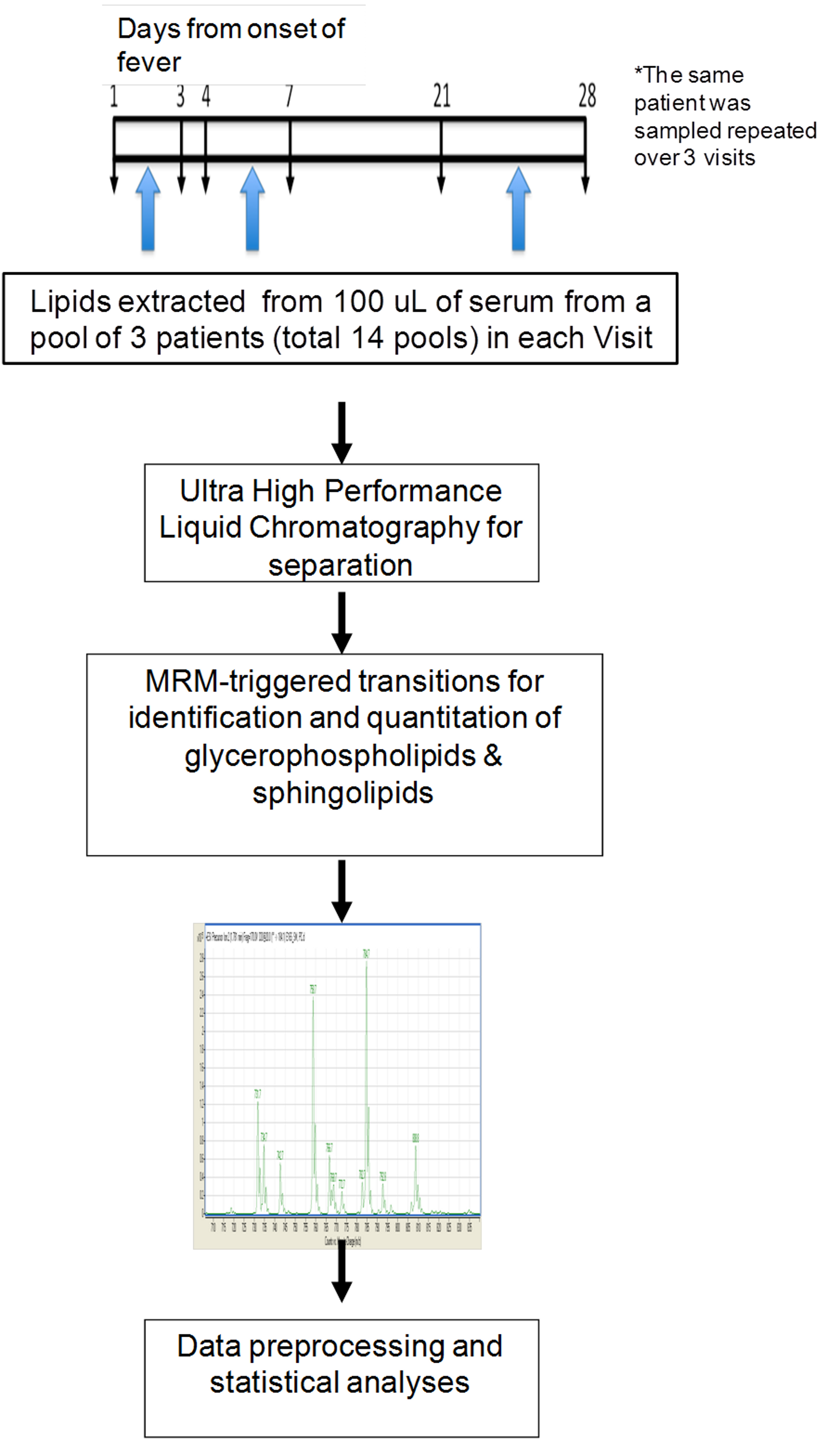

Supplement: Figure S2 — Schematic workflow of mass spectrometry-based lipidomics on serum samples collected from EDEN study. (TIF) [file pntd.0002373.s002.tif]

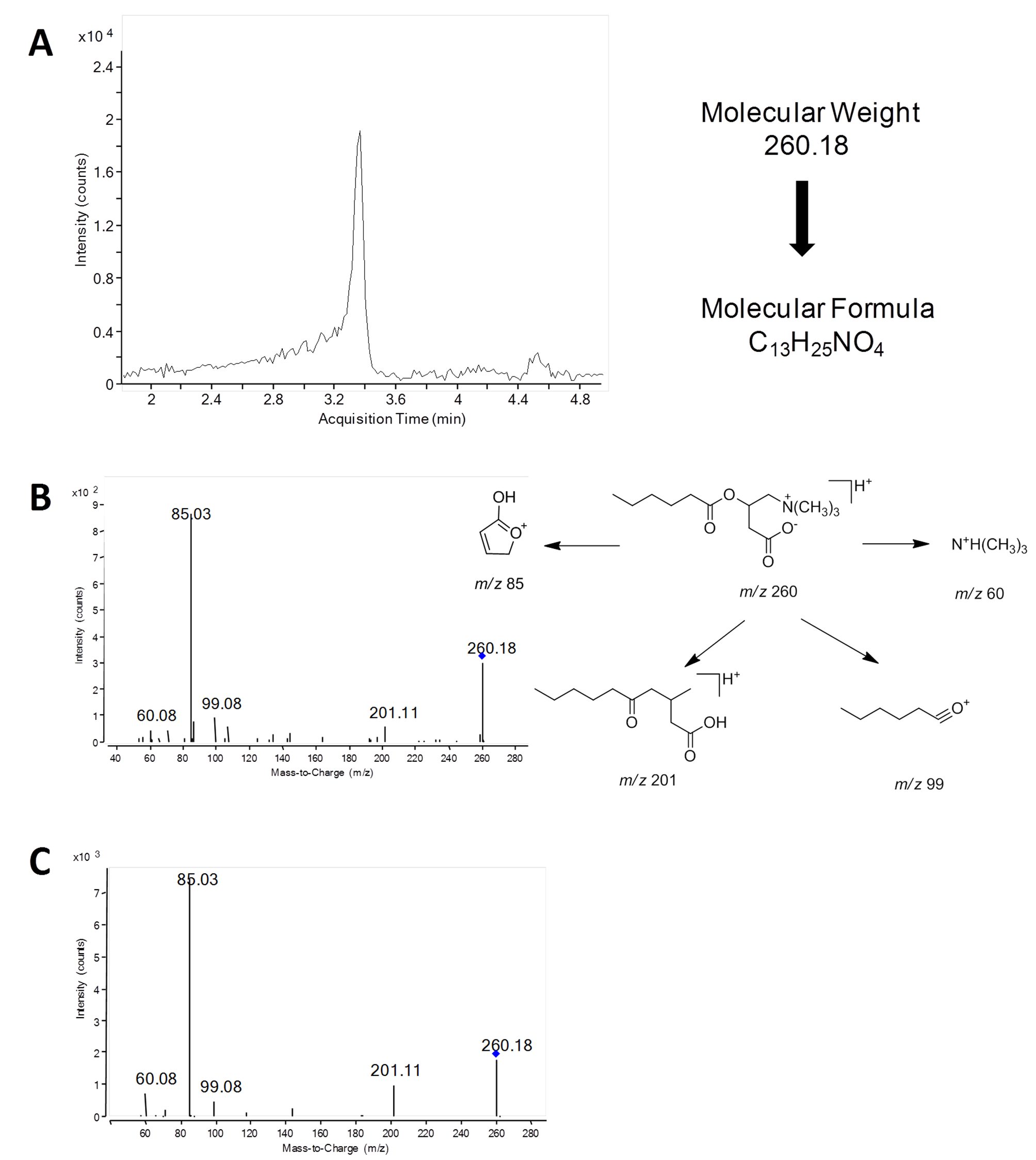

Supplement: Figure S3 — Identification of hexanoylcarnitine as a differential metabolite in dengue fever. A. the extracted ion chromatogram (EIC) and matched formula of the ion m/z 260.18. B. MS/MS spectrum and proposed fragmentation pathways of the ion m/z 260.18. C. MS/MS spectrum of a commercial standard hexanoylcarnitine. (TIF) [file pntd.0002373.s003.tif]

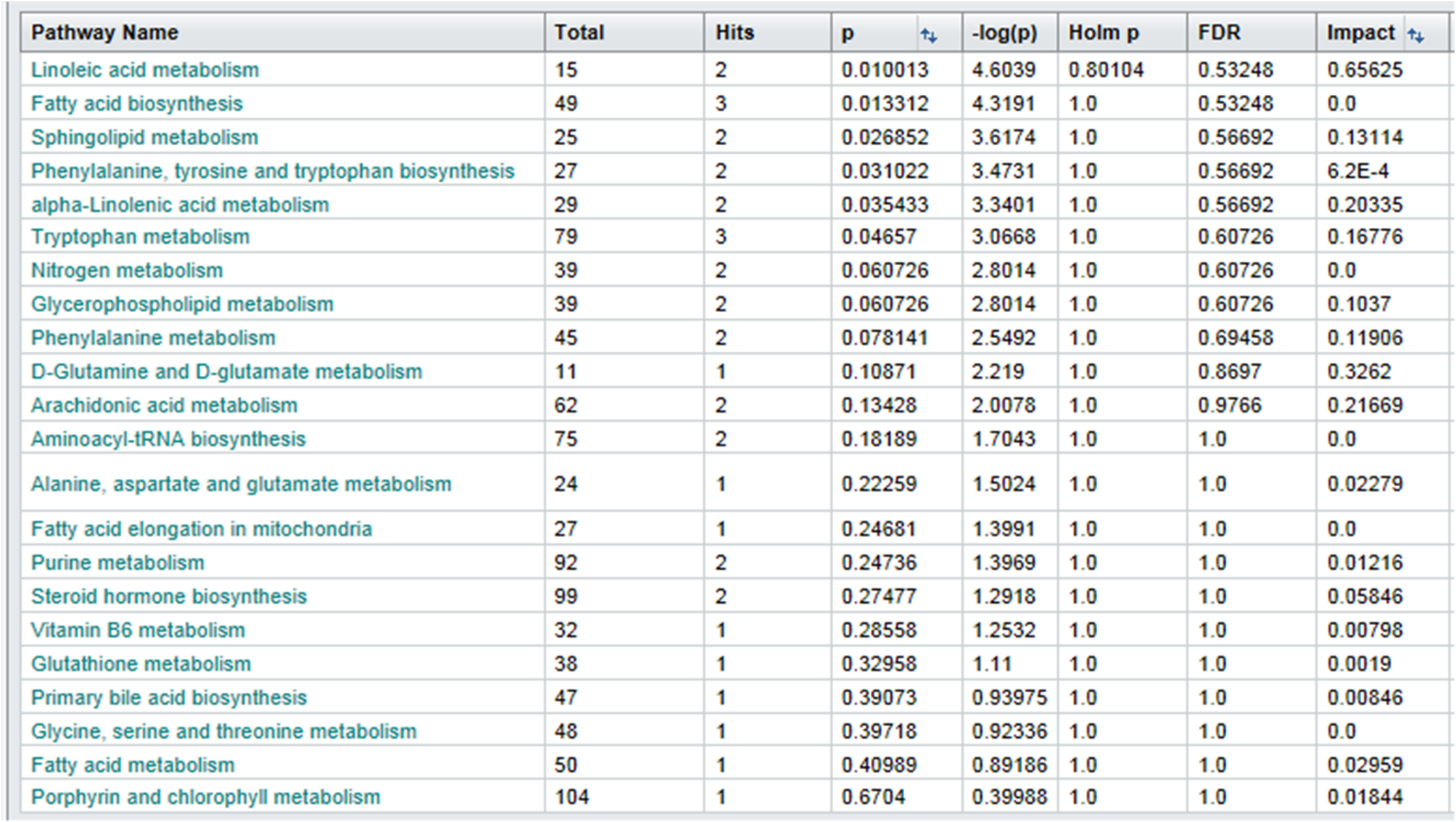

Supplement: Figure S4 — Pathway analysis results by MetaboAnalyst. (TIF) [file pntd.0002373.s004.tif]

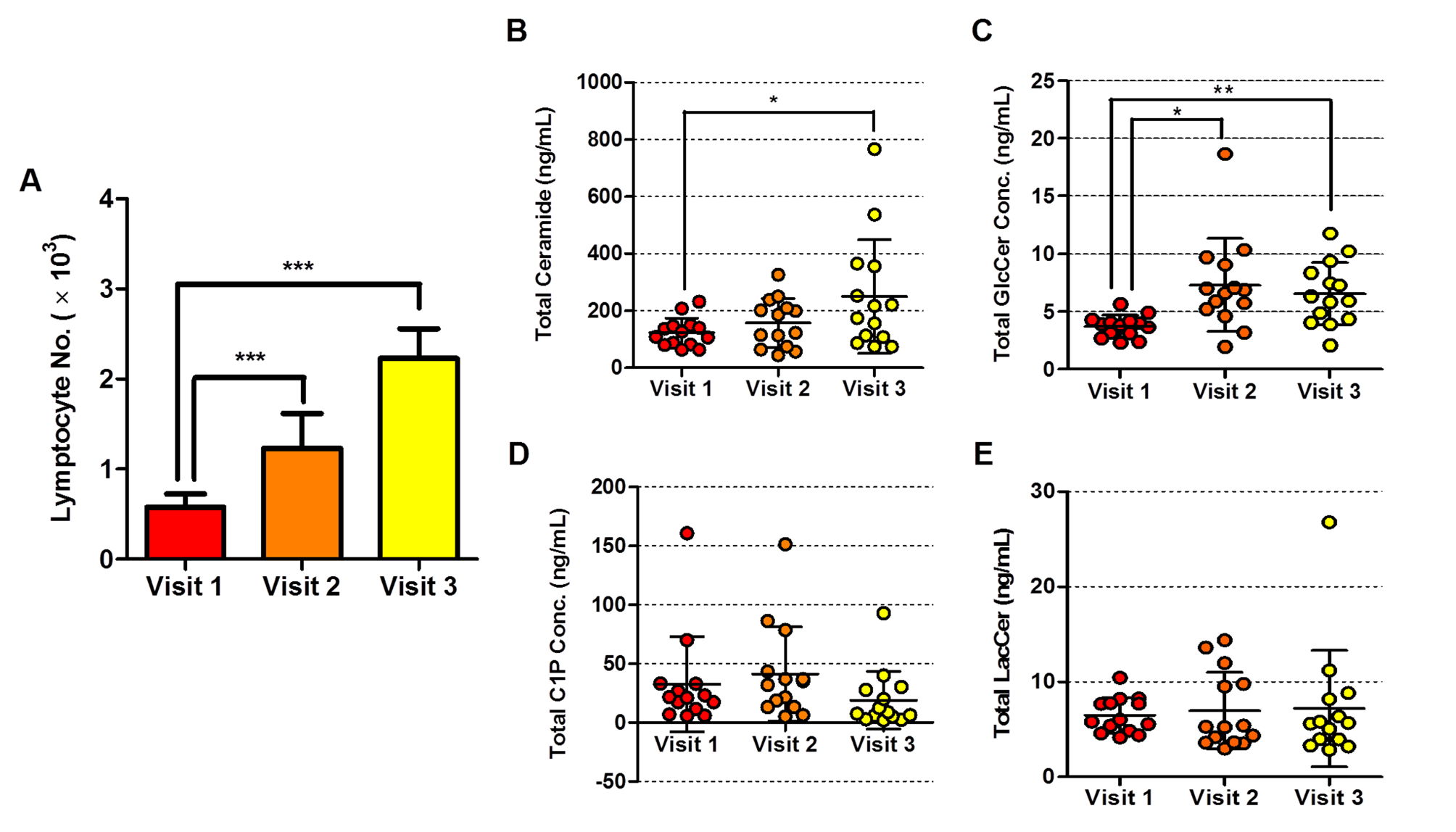

Supplement: Figure S5 — Temporal trends of A. lymphocyte numbers and B. Cer, C. GlcCer, D. C1P and E. LacCer levels. *p<0.05; **p<0.01. (TIF) [file pntd.0002373.s005.tif]

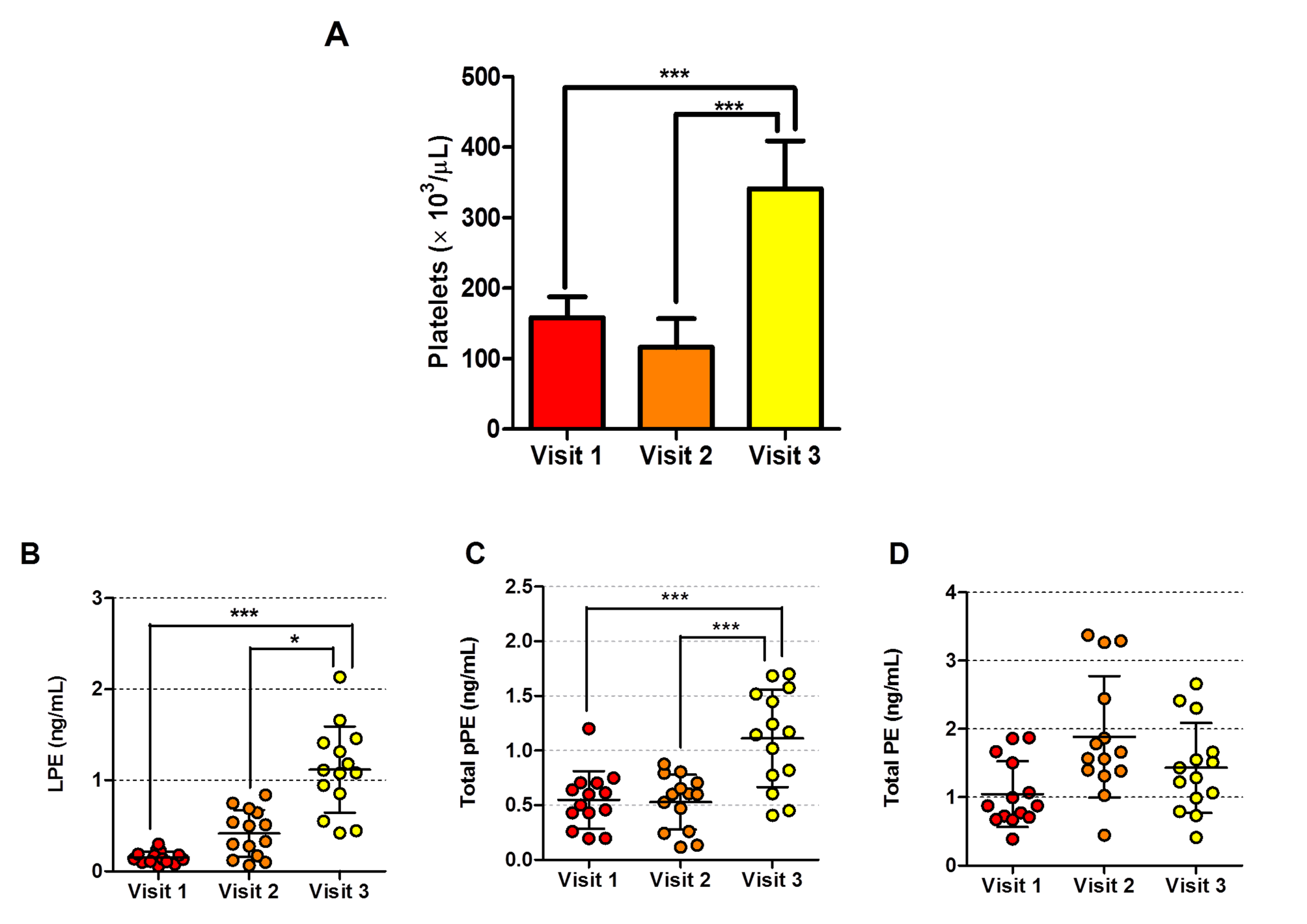

Supplement: Figure S6 — Temporal trends of A. platelet numbers and B. lysoPE, C. pPE and D. PE levels. *p<0.05; ***p<0.0001. (TIF) [file pntd.0002373.s006.tif]

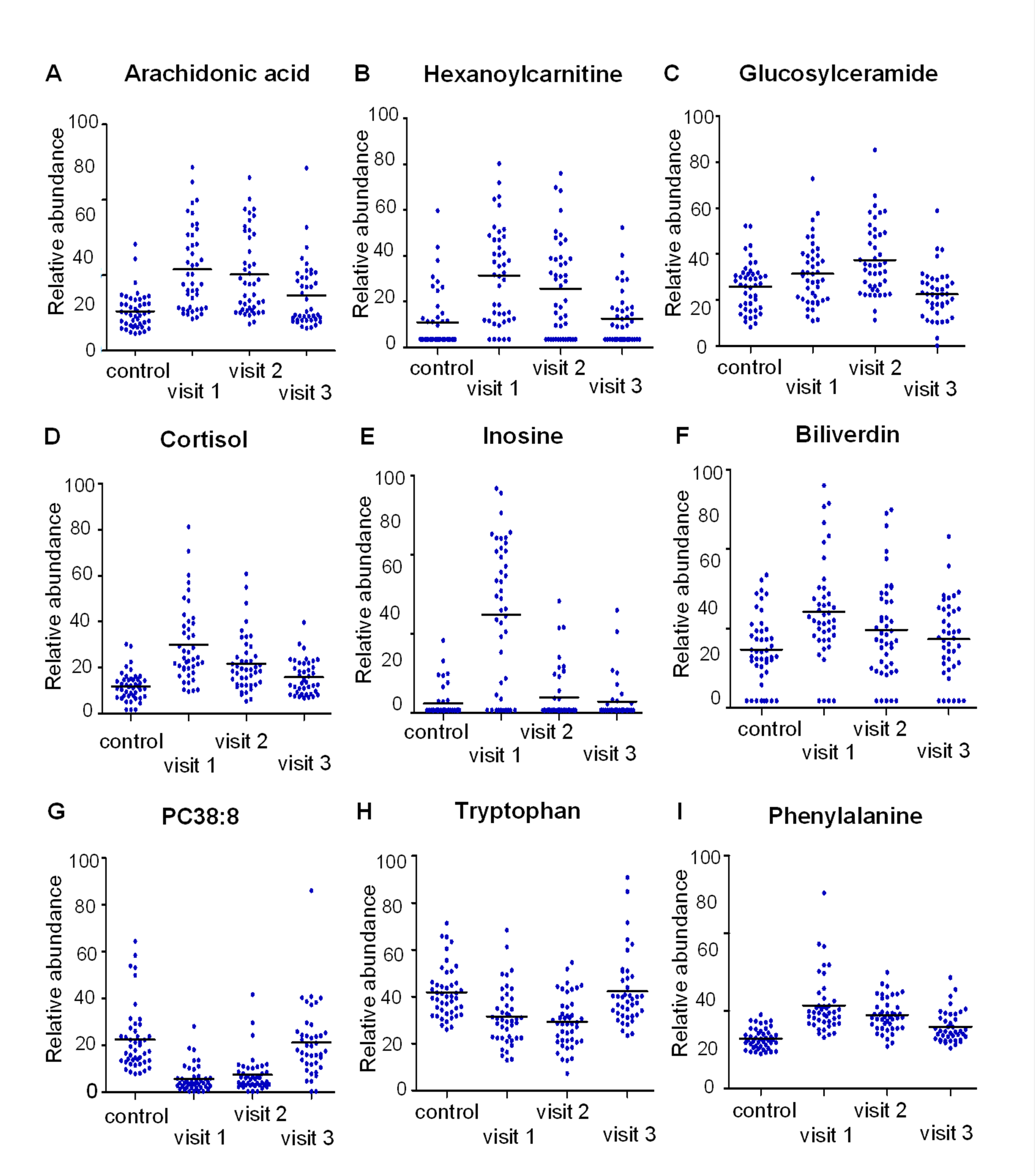

Supplement: Figure S7 — Scatter plots for the major differential metabolite classes. A. arachidonic acid B. hexanoylcarnitine C. glucosylceramide D. cortisol E. inosine F. biliverdine G. PC 38∶8 H. tryptophan I. phenylalanine. The black band near the middle of the plot is the mean peak area of the metabolite. (TIF) [file pntd.0002373.s007.tif]
