# Supplementary material for: Serum Metabolome and Lipidome Changes in Adult Patients with Primary Dengue Infection
Source: PLoS Negl Trop Dis. 2013 Aug 15;7(8):e2373. doi: 10.1371/journal.pntd.0002373 (PMC3744433; doi:10.1371/journal.pntd.0002373)
Supplement: Table S1 — Reproducibility evaluation based on selected peaks in quality control samples. (DOCX) [file pntd.0002373.s008.docx]

**Table S1.** Reproducibility evaluation based on selected peaks in quality control samples.

| **No.** | ***m/z*** | **t_R_ (min)** | **S.D. t_R_^a^(min)** | **RSD^b^ (100%)** |
| --- | --- | --- | --- | --- |
| **1** | 261.13 | 2.1 | 0.003 | 5.2 |
| **2** | 733.33 | 4.0 | 0.007 | 5.6 |
| **3** | 314.23 | 5.9 | 0.01 | 10.6 |
| **4** | 586.27 | 6.3 | 0.007 | 7.1 |
| **5** | 437.19 | 7.4 | 0.003 | 7.4 |
| **6** | 472.30 | 9.0 | 0.009 | 8.8 |
| **7** | 353.26 | 12.2 | 0.017 | 8.3 |
| **8** | 546.35 | 14.0 | 0.011 | 6.5 |

^a^S.D. t_R_: standard deviation of retention times. ^b^RSD: relative standard deviation of peak areas.
