# Supplementary material for: Serum Metabolome and Lipidome Changes in Adult Patients with Primary Dengue Infection
Source: PLoS Negl Trop Dis. 2013 Aug 15;7(8):e2373. doi: 10.1371/journal.pntd.0002373 (PMC3744433; doi:10.1371/journal.pntd.0002373)
Supplement: Table S4 — Identified differential metabolites from LC-MS/MS analysis. (DOCX) [file pntd.0002373.s011.docx]

**Table S3.** Identified differential metabolites from LC-MS/MS analysis.

| **m/z** | **Ion** | **metabolite** | **Chemical formula** | **Fold change*** | **pathway** |
| --- | --- | --- | --- | --- | --- |
| 166.0859 | [M+H]^+^ | L-phenylalanine | C_9_H_11_NO_2_ | +1.66 | Phenylalanine metabolism |
| 205.0972 | [M+H]^+^ | L-Tryptophan | C_11_H_12_N_2_O_2_ | -1.51 | Tryptophan metabolism |
| 130.0497 | [M+H]^+^ | pyroglutamic acid | C_5_H_7_O_3_ | +4.34 | Glutathione metabolism |
| 229.1532 | [M+H]^+^ | L-leucyl-L-proline | C_11_H_20_N_2_O_3_ | +3.32 | Dipeptide |
| 313.1554 | [M+H]^+^ | Phenylalanyl phenylalanine | C_18_H_20_N_2_O_3_ | -2.62 | Dipeptide |
| 188.0705 | [M+H]^+^ | Indoleacrylic acid | C_11_H_9_NO_2_ | -2.00 | Tryptophan metabolism |
| 176.0705 | [M+H]^+^ | Indoleacetic acid | C_10_H_9_NO_2_ | -1.70 | Tryptophan metabolism |
| 137.0448 | [M+H]^+^ | Hypoxanthine | C_5_H_4_N_4_O | +5.95 | Purine metabolism |
| 269.0877 | [M+H]^+^ | Inosine | C_10_H_12_N_4_O_5_ | +6.23 | Purine metabolism |
| 363.2164 | [M+H]^+^ | Cortisol | C_21_H_30_O_5_ | +2.51 | Hormone biosynthesis |
| 583.2551 | [M+H]^+^ | Biliverdin | C_33_H_34_N_4_O_6_ | +1.65 | Heme metabolism |
| 448.3096 | [M-H]^-^ | Glycoursodeoxy-cholic acid | C_26_H_43_NO_5_ | +2.34 | Cholesterol metabolism |
| 464.3051 | [M-H]^-^ | Glycocholic acid | C_26_H_43_NO_6_ | +3.25 | Cholesterol metabolism |
| 187.0080 | [M-H]^-^ | p-Cresol sulfate | C_7_H_8_O_4_S | -3.06 | Cresol metabolism |
| 255.2314 | [M+H]^+^ | Palmitoleic acid | C_16_H_30_O_2_ | +4.10 | Fatty acid synthesis |
| 283.2625 | [M+H]^+^ | Oleic acid | C_18_H_34_O_2_ | +2.66 | Fatty acid synthesis |
| 257.2471 | [M+H]^+^ | Palmitic acid | C_16_H_32_O_2_ | +1.85 | Fatty acid synthesis |
| 281.2465 | [M+H]^+^ | Linoleic acid | C_18_H_32_O_2_ | +2.13 | Arachidonic acid metabolism |
| 329.2464 | [M+H]^+^ | DHA | C_22_H_32_O_2_ | +2.09 | DHA metabolism |
| 305.2468 | [M+H]^+^ | Arachidonic acid | C_20_H_32_O_2_ | +2.07 | Arachidonic acid metabolism |
| 279.2318 | [M+H]^+^ | α-Linoleic acid | C_18_H_30_O_2_ | +2.62 | DHA metabolism |
| 260.1853 | [M+H]^+^ | Hexanoylcarnitine | C_13_H_25_NO_4_ | +2.87 | Lipid *β*-oxidation |
| 368.2786 | [M+H]^+^ | 3, 5-Tetradecadien-carnitine | C_21_H_37_NO_4_ | +3.90 | Lipid *β*-oxidation |
| 370.2939 | [M+H]^+^ | cis-5-Tetrade-cenoylcarnitine | C_21_H_39_NO_4_ | +3.56 | Lipid *β*-oxidation |
| 372.3099 | [M+H]^+^ | Tetradecanoyl-carnitine | C_21_H_41_NO_4_ | +2.22 | Lipid *β*-oxidation |
| 398.3256 | [M+H]^+^ | 9-Hexadecenoyl-carnitine | C_23_H_43_NO_4_ | +3.62 | Lipid *β*-oxidation |
| 426.3578 | [M+H]^+^ | Oleoylcarnitine | C_25_H_47_NO_4_ | +1.52 | Lipid *β*-oxidation |
| 316.2489 | [M+H]^+^ | Decanoylcarnitine | C_17_H_33_NO_4_ | +1.83 | Lipid *β*-oxidation |
| 288.2164 | [M+H]^+^ | L-Octanoylcarnitine | C_15_H_29_NO_4_ | +1.57 | Lipid *β*-oxidation |
| 314.2326 | [M+H]^+^ | 9-Decenoylcarnitine | C_17_H_31_NO_4_ | +1.69 | Lipid *β*-oxidation |
| 344.2797 | [M+H]^+^ | Dodecanoylcarnitine | C_19_H_37_NO_4_ | +2.37 | Lipid *β*-oxidation |
| 722.5538 | [M+Na]^+^ | Glucosylceramide (d18:1/16:0) | C_40_H_77_NO_8_ | +1.54 | Sphingolipid metabolism |
| 729.5822 | [M+H]^+^ | SM(d18:1/18:1(9Z)) | C_41_H_81_N_2_O_6_P | +1.56 | Glycosphingolipid metabolism |
| 355.2843 | [M+H]^+^ | MG(18:2(9Z,12Z)/ 0:0/0:0) | C_21_H_38_O_4_ | +4.17 | Triglyceride metabolism |
| 357.2999 | [M+H]^+^ | MG(18:1(9Z)/ 0:0/0:0) | C_21_H_40_O_4_ | +4.83 | Triglyceride metabolism |
| 617.5146 | [M+H]^+^ | DG(22:4(7Z,10Z, 13Z,16Z)/14:0/0:0) | C_39_H_68_O_5_ | +2.36 | Triglyceride metabolism |
| 468.3087 | [M+H]^+^ | LPC (14:0/0:0) | C_22_H_46_NO_7_P | -2.61 | Phospholipids catabolism |
| 494.3236 | [M+H]^+^ | LPC (0:0/16:1) | C_24_H_48_NO_7_P | -1.96 | Phospholipids catabolism |
| 494.3246 | [M+H]^+^ | LPC (16:1/0:0) | C_24_H_48_NO_7_P | -2.30 | Phospholipids catabolism |
| 496.3394 | [M+H]^+^ | LPC (0:0/16:0) | C_24_H_50_NO_7_P | -1.91 | Phospholipids catabolism |
| 542.3244 | [M+H]^+^ | LPC (0:0/20:5) | C_28_H_48_NO_7_P | -2.46 | Phospholipids catabolism |
| 520.3406 | [M+H]^+^ | LPC (0:0/18:2) | C_26_H_50_NO_7_P | -2.61 | Phospholipids catabolism |
| 520.3398 | [M+H]^+^ | LPC (18:2/0:0) | C_26_H_50_NO_7_P | -2.39 | Phospholipids catabolism |
| 522.3560 | [M+H]^+^ | LPC (0:0/18:1) | C_26_H_52_NO_7_P | -1.67 | Phospholipids catabolism |
| 522.3563 | [M+H]^+^ | LPC (18:1/0:0) | C_26_H_52_NO_7_P | -1.71 | Phospholipids catabolism |
| 524.3716 | [M+H]^+^ | LPC (0:0/18:0) | C_26_H_54_NO_7_P | -1.95 | Phospholipids catabolism |
| 524.3708 | [M+H]^+^ | LPC (18:0/0:0) | C_26_H_54_NO_7_P | -1.84 | Phospholipids catabolism |
| 482.3241 | [M+H]^+^ | LPE 18:0 | C_23_H_48_NO_7_P | -2.44 | Phospholipids catabolism |
| 478.2941 | [M+H]^+^ | LPE 18:2 | C_23_H_44_NO_7_P | -3.11 | Phospholipids catabolism |
| 778.5378 | [M+H]^+^ | PC 36:6 | C_44_H_84_NO_8_P | -2.76 | Phospholipids catabolism |
| 776.5216 | [M+H]^+^ | PC 36:7 | C_44_H_86_NO_8_P | -2.63 | Phospholipids catabolism |
| 780.5538 | [M+H]^+^ | PC 36:5 | C_44_H_82_NO_8_P | -2.41 | Phospholipids catabolism |
| 782.5702 | [M+H]^+^ | PC 36:4 | C_44_H_80_NO_8_P | -3.29 | Phospholipids catabolism |
| 784.5846 | [M+H]^+^ | PC 36:3 | C_44_H_78_NO_8_P | -1.81 | Phospholipids catabolism |
| 804.5546 | [M+H]^+^ | PC 38:7 | C_46_H_78_NO_8_P | -3.95 | Phospholipids catabolism |
| 802.5379 | [M+H]^+^ | PC 38:8 | C_46_H_76_NO_8_P | -3.93 | Phospholipids catabolism |

*The fold change is either visit 1/control or visit 2/control, whichever is larger. +, up-regulation; -, down-regulation.
