# Supplementary material for: Serum Metabolome and Lipidome Changes in Adult Patients with Primary Dengue Infection
Source: PLoS Negl Trop Dis. 2013 Aug 15;7(8):e2373. doi: 10.1371/journal.pntd.0002373 (PMC3744433; doi:10.1371/journal.pntd.0002373)
Supplement: Table S5 — Unidentified differential metabolites from LC-MS/MS analysis. (DOCX) [file pntd.0002373.s012.docx]

**Table S4. Unidentified differential metabolites from LC-MS/MS analysis.**

| **m/z** | **Ion** | **Chemical formula** | **Change trend** | **m/z** | **Ion** | **Chemical formula** | **Change trend** |
| --- | --- | --- | --- | --- | --- | --- | --- |
| 279.0837 | [M+H]^+^ | C12 H13 N3 O5 | ↓ | 312.1615 | [M+H]^+^ | C22 H20 N2 | ↑ |
| 443.8362 | [M+H]^+^ | C15 H3 Cl3 N2 O2 S3 | ↑ | 424.2597 | [M+H]^+^ | C17 H40 N6 O2 S2 | ↑ |
| 385.8876 | [M+H]^+^ | C21 Cl2 S2 | ↑ | 438.2297 | [M+H]^+^ | C18 H31 Cl N10 O | ↑ |
| 165.0809 | [M+H]^+^ | C9 H11 N O2 | ↑ | 316.1715 | [M+H]^+^ | C14 H28 N4 S2 | ↑ |
| 119.075 | [M+H]^+^ | C8 H9 N | ↑ | 622.4477 | [M+H]^+^ | C34 H62 N4 O4 S | ↑ |
| 148.0528 | [M+H]^+^ | C9 H8 O2 | ↑ | 298.1883 | [M+H]^+^ | C15 H27 Cl N4 | ↑ |
| 222.0998 | [M+H]^+^ | C11 H14 N2 O3 | ↓ | 338.1767 | [M+H]^+^ | C24 H22 N2 | ↑ |
| 280.1053 | [M+H]^+^ | C13 H16 N2 O5 | ↓ | 292.1716 | [M+H]^+^ | C11 H24 N4 O5 | ↑ |
| 180.0646 | [M+H]^+^ | C7 H8 N4 O2 | ↑ | 681.3296 | [M+H]^+^ | C34 H51 N O13 | ↓ |
| 304.1495 | [M+H]^+^ | C18 H24 O2 S | ↓ | 743.2862 | [M+H]^+^ | C28 H57 N O15 S3 | ↓ |
| 294.1213 | [M+H]^+^ | C14 H18 N2O5 | ↑ | 340.1780 | [M+H]^+^ | C20 H24 N2 O3 | ↑ |
| 378.1862 | [M+H]^+^ | C13 H26 N6 O7 | ↑ | 318.1878 | [M+H]^+^ | C13 H26 N4 O5 | ↑ |
| 278.1625 | [M+H]^+^ | C15 H22 N2 O3 | ↓ | 682.3773 | [M+H]^+^ | C28 H58 N8 O7 S2 | ↑ |
| 312.1474 | [M+H]^+^ | C18 H20 N2 O3 | ↓ | 262.2292 | [M+H]^+^ | C18 H30 O | ↑ |
| 351.1580 | [M+H]^+^ | C20 H21 N3 O3 | ↓ | 324.2051 | [M+H]^+^ | C18 H24 N6 | ↑ |
| 312.1479 | [M+H]^+^ | C18 H20 N2 O3 | ↓ | 582.4496 | [M+H]^+^ | C30 H59 Cl N8 O | ↑ |
| 748.3751 | [M+H]^+^ | C34 H60 N4 O10 S2 | ↓ | 338.2811 | [M+H]^+^ | C21 H38 O3 | ↑ |
| 320.1233 | [M+H]^+^ | C17 H20 O6 | ↓ | 626.4651 | [M+H]^+^ | C27 H58 N14 O S | ↓ |
| 164.0687 | [M+H]^+^ | C7 H8 N4 O | ↑ | 103.0996 | [M+H]^+^ | C5 H13 N O | ↓ |
| 298.1419 | [M+H]^+^ | C15 H22 O6 | ↑ | 1069.2100 | [M+H]^+^ | C35 H31 N27 O11 S2 | ↓ |
| 1049.4666 | [M+H]^+^ | C44 H79 N3 O21 S2 | ↓ | 1042.6324 | [M+H]^+^ | C54 H98 N4 O7 S4 | ↓ |
| 600.2577 | [M+H]^+^ | C33 H36 N4 O7 | ↑ | 523.3634 | [M+H]^+^ | C22 H45 N13 S | ↑ |
| 863.4024 | [M+H]^+^ | C38 H65 N5 O13 S2 | ↓ | 238.2294 | [M+H]^+^ | C16 H30 O | ↑ |
| 189.0795 | [M+H]^+^ | C11 H11 N O2 | ↑ | 270.2557 | [M+H]^+^ | C17 H34 O2 | ↑ |
| 320.1237 | [M+H]^+^ | C14 H12 N10 | ↑ | 856.6481 | [M+H]^+^ | C46 H92 N6 O2 S3 | ↑ |
| 853.0105 | [M+H]^+^ | C32 H15 N5 O24 | ↑ | 534.3874 | [M+H]^+^ | C28 H58 N2 O3 S2 | ↑ |
| 810.3889 | [M+H]^+^ | C30 H62 N6 O17 S | ↑ | 242.2226 | [M+H]^+^ | C15 H30 O2 | ↓ |
| 810.1891 | [M+H]^+^ | C50 H34 O11 | ↑ | 465.3212 | [M+H]^+^ | C24 H43 N5 O4 | ↓ |
| 414.2045 | [M+H]^+^ | C25 H26 N4 O2 | ↑ | 440.2869 | [M+H]^+^ | C22 H46 Cl2 N2 O2 | ↓ |
| 518.3404 | [M+H]^+^ | C24 H50 N6 O2 S2 | ↓ | 424.3155 | [M+H]^+^ | C25 H44 O5 | ↓ |
| 299.2820 | [M+H]^+^ | C18 H37 N O2 | ↑ | 632.1414 | [M+H]^+^ | C30 H25 Cl N6 O8 | ↓ |
| 488.3311 | [M+H]^+^ | C26 H44 N6 O S | ↓ | 462.3316 | [M+H]^+^ | C23 H47 Cl N4 O3 | ↑ |
| 467.3008 | [M+H]^+^ | C22 H49 N3 O S3 | ↓ | 537.3834 | [M+H]^+^ | C27 H59 N3 O S3 | ↓ |
| 557.2707 | [M+H]^+^ | C22 H43 N3 O13 | ↓ | 264.2451 | [M+H]^+^ | C18 H32 O | ↑ |
| 517.3161 | [M+H]^+^ | C29 H47 N3 O S2 | ↓ | 324.2030 | [M+H]^+^ | C22 H28 O2 | ↑ |
| 625.2693 | [M+H]^+^ | C33 H39 N9 S2 | ↓ | 618.4636 | [M+H]^+^ | C42 H63 Cl O | ↑ |
| 648.4398 | [M+H]^+^ | C34 H68 N2 O3 S3 | ↓ | 678.5093 | [M+H]^+^ | C37 H74 O8 S | ↑ |
| 622.3493 | [M+H]^+^ | C35 H58 O3 S3 | ↓ | 684.5246 | [M+H]^+^ | C35 H72 N8 O S2 | ↑ |
| 739.2584 | [M+H]^+^ | C27 H33 N17 O9 | ↓ | 746.496 | [M+H]^+^ | C40 H70 N6 O3 S2 | ↑ |
| 477.2856 | [M+H]^+^ | C23 H39 N7 O2 S | ↓ | 606.2439 | [M+H]^+^ | C35 H34 N4 O6 | ↓ |
| 599.2709 | [M+H]^+^ | C37 H42 Cl N O4 | ↓ | 330.2526 | [M+H]^+^ | C22 H34 O2 | ↑ |
| 103.0999 | [M+H]^+^ | C5 H13 N O | ↑ | 346.2182 | [M+H]^+^ | C15 H30 N4 O8 | ↑ |
| 646.4308 | [M+H]^+^ | C29 H62 N10 S3 | ↓ | 551.3913 | [M+H]^+^ | C32 H57 N O4 S | ↓ |
| 670.4314 | [M+H]^+^ | C32 H60 Cl2 N10 O | ↓ | 311.2821 | [M+H]^+^ | C19 H37 N O2 | ↓ |
| 1034.5612 | [M+H]^+^ | C35 H66 N30 O8 | ↓ | 450.3655 | [M+H]^+^ | C27 H50 N2 O S | ↓ |
| 1018.5934 | [M+H]^+^ | C56 H86 N6 O7 S2 | ↓ | 668.4844 | [M+H]^+^ | C28 H60 N16 O S | ↓ |
| 1080.6274 | [M+H]^+^ | C51 H88 N18 S4 | ↓ | 578.4521 | [M+H]^+^ | C36 H66 O S2 | ↑ |
| 946.5998 | [M+H]^+^ | C53 H86 O12 S | ↓ | 408.3364 | [M+H]^+^ | C29 H44 O | ↑ |
| 579.2852 | [M+H]^+^ | C26 H45 N O13 | ↓ | 600.4701 | [M+H]^+^ | C33 H65 Cl N4 O3 | ↑ |
| 715.2583 | [M+H]^+^ | C31 H45 N3 O14 S | ↑ | 922.4912 | [M+H]^+^ | C46 H74 N4 O13 S | ↓ |
| 1038.6584 | [M+H]^+^ | C43 H86 N22 O4 S2 | ↓ | 875.5402 | [M+H]^+^ | C43 H77 N11 O2 S3 | ↓ |
| 585.3018 | [M+H]^+^ | C24 H43 N9 O6 S | ↓ | 477.3224 | [M+H]^+^ | C27 H39 N7 O | ↓ |
| 1062.6572 | [M+H]^+^ | C43 H87 Cl N20 O9 | ↑ | 895.5268 | [M+H]^+^ | C38 H73 N17 O2 S3 | ↓ |
| 328.2126 | [M+H]^+^ | C21 H28 O3 | ↑ | 743.5436 | [M+H]^+^ | C39 H77 N5 O4 S2 | ↓ |
| 266.1554 | [M+H]^+^ | C13 H22 N4 S | ↑ | 781.5622 | [M+H]^+^ | C40 H83 N3 O7 S2 | ↓ |
| 298.1819 | [M+H]^+^ | C11 H22 N8 O2 | ↑ | 559.4917 | [M+H]^+^ | C32 H69 N3 S2 | ↑ |
| 827.5441 | [M+H]^+^ | C37 H61 N23 | ↑ | 369.1765 | [M+H]^+^ | C19 H23 N5 O3 | ↓ |
| 763.5154 | [M+H]^+^ | C43 H69 N7 O3 S | ↓ | 387.3861 | [M+H]^+^ | C27 H49 N | ↓ |
| 699.5178 | [M+H]^+^ | C42 H73 N3 O S2 | ↓ | 406.2329 | [M+H]^+^ | C23 H34 O6 | ↓ |
| 725.5333 | [M+H]^+^ | C44 H75 N3 O S2 | ↑ | 374.2956 | [M+H]^+^ | C22 H46 S2 | ↓ |
| 632.4765 | [M+H]^+^ | C35 H72 N2 O S3 | ↑ | 450.2951 | [M+H]^+^ | C20 H38 N10 S | ↓ |
| 616.5037 | [M+H]^+^ | C32 H68 N6 O3 S | ↑ | 342.1935 | [M+H]^+^ | C20 H26 N2 O3 | ↑ |
| 151.0635 | [M+H]^+^ | C8 H9 N O2 | ↑ | 501.3218 | [M+H]^+^ | C26 H51 N3 S3 | ↓ |
| 1072.4072 | [M+H]^+^ | C47 H48 N26 O4 S | ↓ | 668.3314 | [M+H]^+^ | C26 H56 N10 S5 | ↑ |
| 562.1920 | [M+H]^+^ | C20 H40 Cl2 N6 O4 S2 | ↑ | 312.0774 | [M+H]^+^ | C14 H20 N2 S3 | ↑ |
| 341.2560 | [M+H]^+^ | C15 H31 N7 O2 | ↑ | 440.1256 | [M+H]^+^ | C20 H28 N2 O3 S3 | ↑ |
| 613.1727 | [M+H]^+^ | C38 H23 N5 O4 | ↑ | 575.4618 | [M+H]^+^ | C33 H69 N S3 | ↑ |
| 196.1460 | [M+H]^+^ | C12 H20 O2 | ↑ | 320.1229 | [M+H]^+^ | C15 H25 Cl O3 S | ↑ |
| 540.1128 | [M+H]^+^ | C32 H25 Cl O4 S | ↑ | 741.2951 | [M+H]^+^ | C34 H51 N3 O11 S2 | ↓ |
| 519.4987 | [M+H]^+^ | C34 H65 N O2 | ↑ | 359.3546 | [M+H]^+^ | C25 H45 N | ↓ |
| 748.3741 | [M+H]^+^ | C34 H60 N4 O10 S2 | ↓ |  |  |  |  |
